# Design and catalytic performance of a novel two-dimensional copper metal-organic framework for green synthesis of tetrahydrobenzo[b]pyrans

**DOI:** 10.1038/s41598-025-14653-1

**Published:** 2025-10-03

**Authors:** Ehsan Joukar Bahaderani, Khosro Mohammadi, Payam Hayati, Jan Janczak

**Affiliations:** 1 Chemistry Department, Faculty of Nano and Bio Sciences and Technology, Persian Gulf University, Bushehr, 75169 Iran; 2 Department of Biotechnology and Life Sciences, University of Insubria, Via Jean Henry Dunant, 3, Varese, 21100 Italy; 3 Institute of Low Temperature and Structure Research, Polish Academy of Sciences, Okólna 2 Str, P. O. Box 1410, Wroclaw, 50-422 Poland

**Keywords:** Metal-organic framework, Copper, Phosphonocarboxylic acid, Nanocatalyst, Tetrahydrobenzo[b]pyran, Multicomponent reaction, Ultrasonic, Chemistry, Nanoscience and technology

## Abstract

This study explores development and using a novel two-dimensional metal-organic framework (2D MOF) as a heterogeneous catalyst in organic synthesis. The phosphonocarboxylic acid *m*-PO_3_CH_2_C_6_H_4_CO_2_H (MPB) has been employed in synthesizing a metal carboxyarylphosphonate. The 2D MOF [Cu(H_2_O)(*m*-PO_3_CH_2_C_6_H_4_CO_2_H)]_n_ was synthesized in both crystal (Cu-MPB) and nanostructure (Cu-MPB′) forms, characterized by X-ray single crystallography, SEM, TGA, FT-IR, PXRD, EDX, Mapping, and nitrogen adsorption-desorption, and employed for the efficient green synthesis of tetrahydrobenzo[b]pyrans. The crystal structure features alternating inorganic and organic layers. The organic layers have supramolecular dimers of carboxylic acid groups, two molecules bonded to two inorganic layers. Tetrahydrobenzo[b]pyran yield optimization conditions were determined with water/ethanol as solvents, using a catalyst loading of 0.06 g for 95% yields. The fabricated catalyst exhibited exceptional catalytic performance, wide applicability to different aldehyde substrates, and remarkable reusability, achieving high yields for as many as seven cycles post-reaction. In comparison to earlier catalytic systems, the compound Cu-MPB′ showed enhanced efficiency and recyclability. These results underscore the promise of this innovative 2D MOF in promoting sustainable and effective organic synthesis.

**Supplementary Information:**

The online version contains supplementary material available at 10.1038/s41598-025-14653-1.

## Introduction

The growing demand for efficient, sustainable, and environmentally friendly catalytic systems in organic synthesis has led to increased interest in metal-organic frameworks (MOFs) as promising heterogeneous catalysts^1,2^. MOFs, composed of metal ions coordinated to organic ligands, offer several distinctive advantages, including high surface areas, tunable porosity, and customizable chemical functionalities^3,4^. These attributes allow MOFs to effectively facilitate various chemical transformations by promoting reactant diffusion, stabilizing transition states, and supporting active catalytic sites^5,6^.

Among MOFs, two-dimensional (2D) structures have emerged as especially promising due to their ultrathin morphology, higher specific surface area, and abundance of unsaturated metal sites^7–9^. These features enhance catalytic efficiency in electrocatalysis, photocatalysis, and thermocatalysis by improving reactant accessibility and promoting charge separation^10,11^. Moreover, 2D MOFs demonstrate thermal and chemical stability comparable to or exceeding their three-dimensional (3D) counterparts^12^. Recent synthetic advancements focus on the strategic design of metal nodes and organic linkers, alongside post-synthetic modifications. Their planar architecture also provides an ideal platform for hosting single-atom catalysts, metal nanoparticles, and molecular catalysts^13^. Notable examples such as (NH_4_)_3_[In_3_Cl_2_(BPDC)_5_]^14^, [Cd(PBA)(DMF)].DMF^15^, and Pd/NUS-SO_3_H^16^ exemplify the versatility and catalytic potential of 2D MOF-based materials. This emphasis on 2D forms underscores the broader significance of nanostructured coordination polymers (CPs), including MOFs, where nanoscale dimensions enhance reactivity and tune physicochemical properties while retaining structural integrity^17,18^. However, achieving controlled synthesis of nanostructured CPs encompassing both 2D MOFs and other nanoscale CP architectures remains challenging due to the acute sensitivity of self-assembly processes to reaction conditions (e.g., solvent composition, metal-ligand ratios, temperature)^19^. Conventional methods often necessitate extended reaction times and harsh conditions^20^, limiting scalable production. In contrast, sonochemical synthesis offers a rapid, energy-efficient alternative for nanoscale CP fabrication, enabling accelerated crystallization and uniform nucleation, particularly advantageous for engineering advanced catalysts such as 2D MOFs^21^.

One of the important target compounds in pharmaceutical and medicinal chemistry is tetrahydrobenzo[b]pyrans, which exhibit a wide range of biological activities including antibacterial, anticancer, and anti-inflammatory effects^22,23^. These compounds have also shown potential in treating neurodegenerative and cognitive disorders such as Alzheimer’s disease and Parkinson’s disease^24^. Despite their importance, conventional synthetic methods for tetrahydrobenzo[b]pyrans often involve multistep procedures, harsh reaction conditions, and the use of toxic reagents, leading to low efficiency and environmental concerns^25^.

Multicomponent reactions (MCRs), which combine three or more reactants in a single step, offer an efficient route for synthesizing these bioactive molecules^26–28^. Although various catalytic systems have been explored for MCR-based synthesis of tetrahydrobenzo[b]pyrans, many suffer from limitations such as high catalyst loading, non-recyclability, and reliance on toxic solvents or high temperatures^29^. Previously reported catalysts include Fe₃O₄@Al₂O₃@[PBVIm]HSO₄^30^, Na₃[CrMo₆O₂₄H₆]0.8 H₂O@ZrO₂^31^, Fe3O4@GA@Isinglass^32^, and MNPs@Alg-Pr-His^33^, yet there remains a pressing need for more sustainable and efficient catalytic approaches.

In this context, the present study addresses these challenges by developing a novel 2D MOF synthesized via a sonochemical method, specifically designed as a heterogeneous catalyst for the green and efficient synthesis of tetrahydrobenzo[b]pyrans. This work aims to bridge gaps in current catalytic systems by integrating the structural benefits of 2D MOFs with the process advantages of sonochemistry, contributing to the advancement of environmentally benign methodologies in organic synthesis.

To the best of our knowledge, this is the first report on the ultrasonic synthesis of a two-dimensional copper-based metal-organic framework (Cu-MPB′) using the phosphonocarboxylic acid ligand MPB, designed specifically as a heterogeneous catalyst for the green and efficient synthesis of tetrahydrobenzo[b]pyrans. This work combines a rapid and mild sonochemical synthesis strategy with the catalytic advantages of a 2D MOF architecture, achieving high yield, excellent recyclability, and performance under environmentally friendly conditions. The multifunctionality and structural stability of the synthesized Cu-MPB′ distinguish it from previously reported systems, advancing the design of sustainable nanocatalysts for multicomponent reactions.

## Experimental section

### Materials and methods

All the chemicals and solvents were purchased from Sigma-Aldrich and Merck and used without further purification. The spectroscopic characterization of the synthesized compounds is achieved by recording Fourier transform infrared (FT-IR) spectra using a JASCO FT/IR-4600 spectrometer. Powder X-ray diffraction (PXRD) was performed using an X’pert diffractometer created by Philips with monochromatized Cu K*α* radiation, and a scanning electron microscope (SEM) was performed using a VEGA3 microscope. Sonochemical experiments were performed using a US Scientz-750 F generator. Melting points were determined using a Krüss KSB1N apparatus in open capillary tubes. Energy-dispersive X-ray spectroscopy (EDX) was performed using an EDAX-EDS Tescan-Vega2 instrument. Thermal gravimetric analysis (TGA) was carried out using a Perkin Elmer STA6000 apparatus. Brunauer-Emmett-Teller (BET) analysis was performed via the Micromeritics ASAP2020 apparatus. The SIGMA 2- 16p centrifuge was effectively utilized in the process of separating catalysts from the mixtures. Nuclear magnetic resonance (NMR) spectra were measured in CDCl_3_ with a (300 MHz) Bruker Avance III NMR system, and chemical shifts are reported in *δ* (ppm) values. Thin layer chromatography (TLC) was performed using Silica gel 60 F254 plates (Merck).

The intensity of single-crystal X-ray data for [Cu(H_2_O)(*m*-PO_3_CH_2_C_6_H_4_CO_2_H)]_n_ (Cu-MPB) was measured using a Xcalibur four-circle κ geometry diffractometer with an Atlas two-dimensional area CCD detector at room temperature. The measurements were performed using graphite monochromatic Mo Kα radiation. Data collections, cell refinements, integration, correction for Lorenz and polarization effects, and absorption corrections were performed using the CrysAlisPro 1.171.42.93a program system^34^. Using Olex2^35^, the structure was solved by the direct methods using SHELXT^36^ and refined with the SHELXL-2018/3 program^37^. The hydrogen atoms joined to aromatic carbon atoms were introduced in their geometrical positions and treated as rigid with U_iso_=1.2U_eq_ (C). The positions of the hydrogen atoms linked to oxygen atoms were refined with U_iso_=1.5U_eq_ (O). The final difference Fourier maps showed no peaks of chemical significance. Details of the data collection parameters, crystallographic data, and final agreement parameters are collected in Table S1. The Diamond 3.0 program^38^ was used for visualization of the structure.

## Synthesis of the single crystal (Cu-MPB) and nanoparticles (Cu-MPB′)

### Synthesis of [Cu(H_2_O)(m-PO_3_CH_2_C_6_H_4_CO_2_H)]_n_ as MOF single crystals (Cu-MPB)

To synthesize crystalline [Cu(H_2_O)(*m*-PO_3_CH_2_C_6_H_4_CO_2_H)]_n_, CuSO_4_.5H_2_O (0.1 mmol, 0.0249 g) and *m*-(phosphonomethyl)benzoic acid (MPB) (0.1 mmol, 0.0216 g) were mixed in 10 mL of methanol and 1 mL deionized water and stirred at ambient temperature for 30 min. A Parr Teflon container was used to hold the reaction mixture, which was subsequently placed inside a stainless-steel tank. Following that, the tank was sealed in the autoclave and heated to 110 °C for a whole day. The reaction mixture was slowly cooled to room temperature, and blue-green crystals (Yield: 18.3%) of [Cu(H_2_O)(*m*-PO_3_CH_2_C_6_H_4_CO_2_H)]_n_ appeared and were identified using X-ray analysis.

### Sonochemical synthesis of [Cu(H_2_O)(m-PO_3_CH_2_C_6_H_4_CO_2_H)]_n_ as MOF nanostructure (Cu-MPB′)

Compound Cu-MPB′ in powder form, [Cu(H_2_O)(*m*-PO_3_CH_2_C_6_H_4_CO_2_H)]_n_, was synthesized by combining CuSO_4_.5H_2_O (1 mmol, 0.249 g) with MPB (1 mmol, 0.216 g) in a solution of 20 mL methanol and 2 mL deionized water. At ambient temperature, the mixture was agitated for thirty minutes. Specifically, the mixture was contained in a beaker and subjected to an ultrasonic frequency of 60 Hz for 30 min. The temperature of the bath was consistently maintained at 50 °C throughout the entire irradiation process. After sonication, a light blue-green powder precipitate was noted, signifying the successful formation of the intended product. The solid was separated through filtration and subsequently purified by rinsing with methanol, followed by extensive washing with deionized water. The resultant light blue-green powder (Yield: 76.1%) was analyzed using suitable analytical methods to verify its identity.

## General procedure for the synthesis of tetrahydrobenzo[b]pyrans derivatives

This process involved the combination of the nanocatalyst Cu-MPB′ (0.06 g) in distilled water/ethanol (5 mL/5 mL), in addition to benzaldehyde (or its derivatives) (1 mmol), malononitrile (1.2 mmol), and dimedone (1 mmol). The mixture was then subjected to vigorous agitation at a temperature of 35 °C. The progress of the reaction was monitored through thin-layer chromatography (TLC). Upon completion of the reaction, the Cu-MPB′ catalyst was separated via centrifugation, followed by the addition of ethanol and subsequent drying. Finally, the solution was cooled in an ice bath to promote the crystallization or precipitation of the desired pure products. The spectroscopic data of the tetrahydrobenzo[b]pyrans derivatives are shown in supporting information section.

## Results and discussion

### Synthetic perspective

**Fig. 1:**
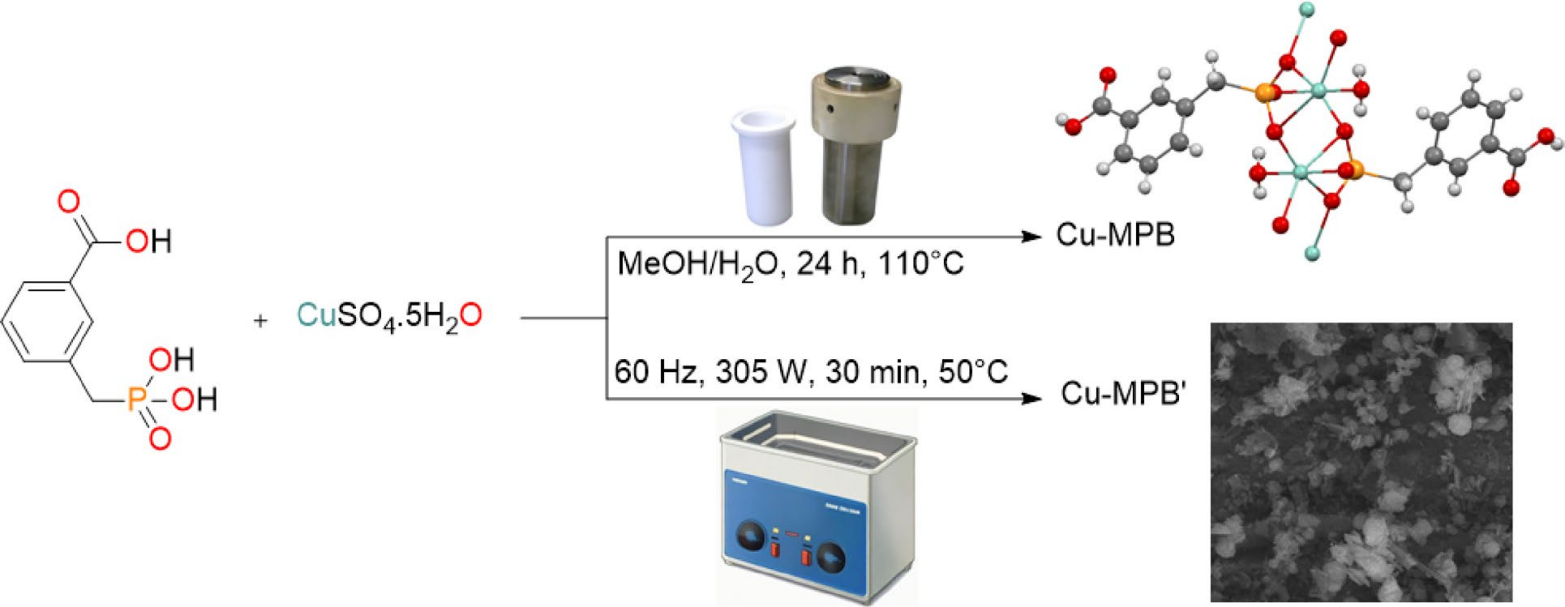
The synthetic routes of compounds Cu-MPB and Cu-MPB′.

The coordination complex, Cu-MPB was synthesized by combining MPB with copper(II) sulfate pentahydrate in a methanol/water solvent system. The solvothermal method was utilized to cultivate single crystals of this novel metal-organic framework, which were appropriate for X-ray crystallographic examination. Additionally, the compound Cu-MPB′ was produced through the reaction of MPB and copper(II) sulfate pentahydrate in a 1:1 stoichiometric ratio within the methanol/water mixture, aided by ultrasonic waves, as depicted in Fig. 1.

### X-ray single crystal structure

#### Crystal structure description

**Fig. 2:**
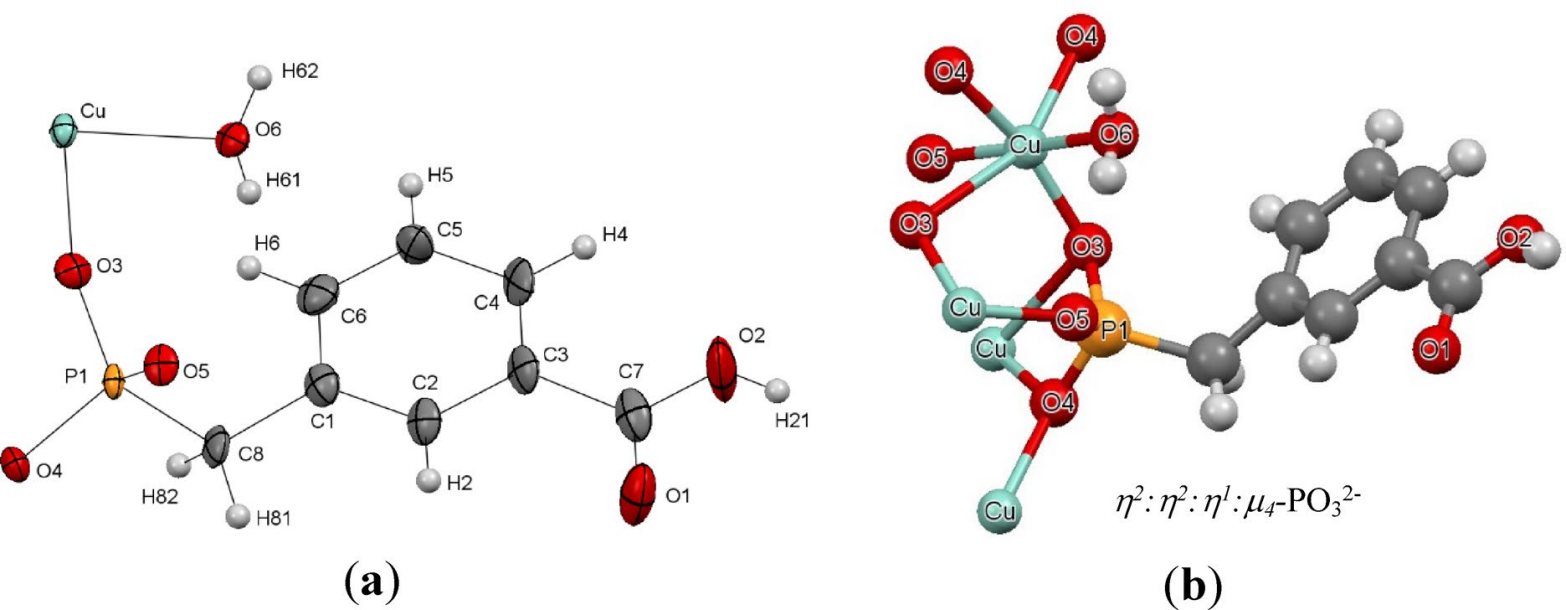
**(a)** ORTEP representation of the asymmetric unit and (**b**) Coordination modes of the MPB linker in Cu-MPB.

The monoclinic centrosymmetric space group P2_1_/n was the crystallization structure for, as shown in Table S1. The ORTEP view of Cu-MPB is depicted in Fig. 2a. Compound Cu-MPB exhibited a unit cell volume of 943.099 Å^3^. Examination of the inorganic layer revealed that Cu^2+^ ions establish connections with the oxygen atoms of the phosphonate groups (Fig. 2b). Water molecules were incorporated to finalize the coordination polyhedron encircling the copper atoms, leading to an octahedral configuration of the oxygen atoms. This octahedron displayed an irregular form, with five of its bonds ranging from 1.938(4) Å to 2.231(4) Å, while one bond was notably extended at 2.581(4) Å. The tetrahedral arrangement around each phosphorus atom is composed of three oxygen atoms and one carbon atom. Each octahedron is connected at its corners to four adjacent octahedra, forming a structure resembling a perovskite layer. In the structure, the PO_3_C tetrahedra are connected to the CuO_6_ octahedra by sharing one edge, forming the CuPO_7_C groups. These groups bear resemblance to the VPO_8_ units found in Li_2_VO_2_PO_4_ frameworks^39^ and layered M(II)(RPO_3_).H_2_O^40^. Furthermore, the PO_3_C tetrahedra shared a single corner with an additional octahedron. The perovskite layer undergoes significant deformation owing to this bonding, causing the octahedra to tilt dramatically in relation to the plane containing Cu^2+^ ions.

**Fig. 3:**
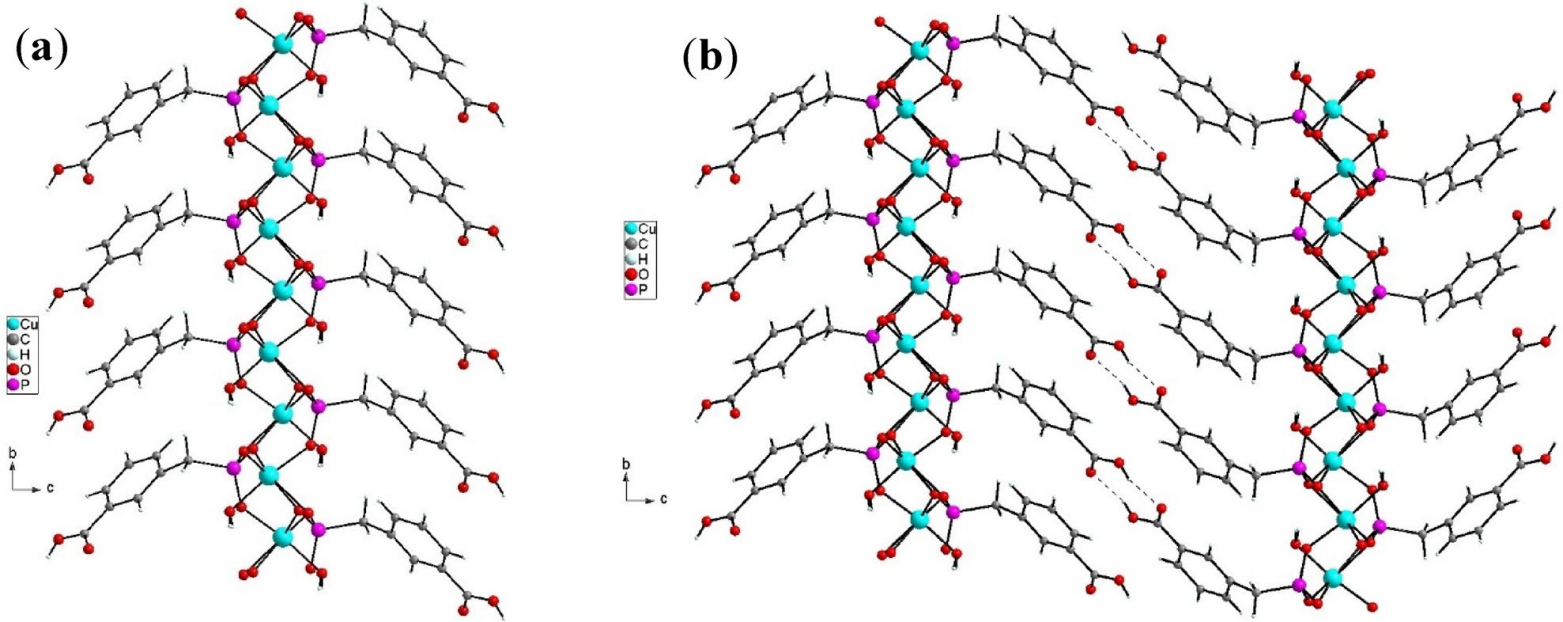
The inter and intramolecular interactions in compound Cu-MPB. **(a**) The covalence and (**b**) the hydrogen bonding.

The Cu-MPB compound exhibits a robust molecular architecture, consisting of alternating layers of inorganic and organic materials systematically arranged (see Fig. 3). The copper ions and ligand units are covalently linked within a two-dimensional framework (refer to Fig. 3a). Additionally, significant hydrogen bonding interactions are present in the three-dimensional structure. The organic component reveals supramolecular dimeric formations characterized by pairs of carboxylic acid groups (illustrated in Fig. 3b). The O1‧‧‧O2 interaction measures 2.614(8) Å, while the O2–H21‧‧‧O1i bond forms an angle of 161°. Furthermore, Table 1 provides a comprehensive overview of the hydrogen bonding interactions involving water molecules that are coordinated to the copper atom.

**Table 1:** Hydrogen-bond data (Å, º) in Cu-MPB.

D—H···A	D—H	H···A	D···A	D—H···A
O2—H21···O1^*i*^	0.82	1.82	2.614 (8)	161
O6—H61···O3^*ii*^	0.90	2.11	2.901 (6)	146
O6—H61···O5	0.90	2.31	2.908 (6)	124
O6—H62···O4^*iii*^	0.90	2.20	3.068 (6)	161
O6—H62···O5^*iv*^	0.90	2.36	2.905 (6)	119

Symmetry codes: (*i*) −*x*+1, −*y*, −*z*+1; (*ii*) *x*, *y*−1, *z*; (*iii*) *x*−1, *y*−1, *z*; (*iv*) *x*−1, *y*, *z*.

### Characterization of nanoparticle Cu-MPB′

**Fig. 4:**
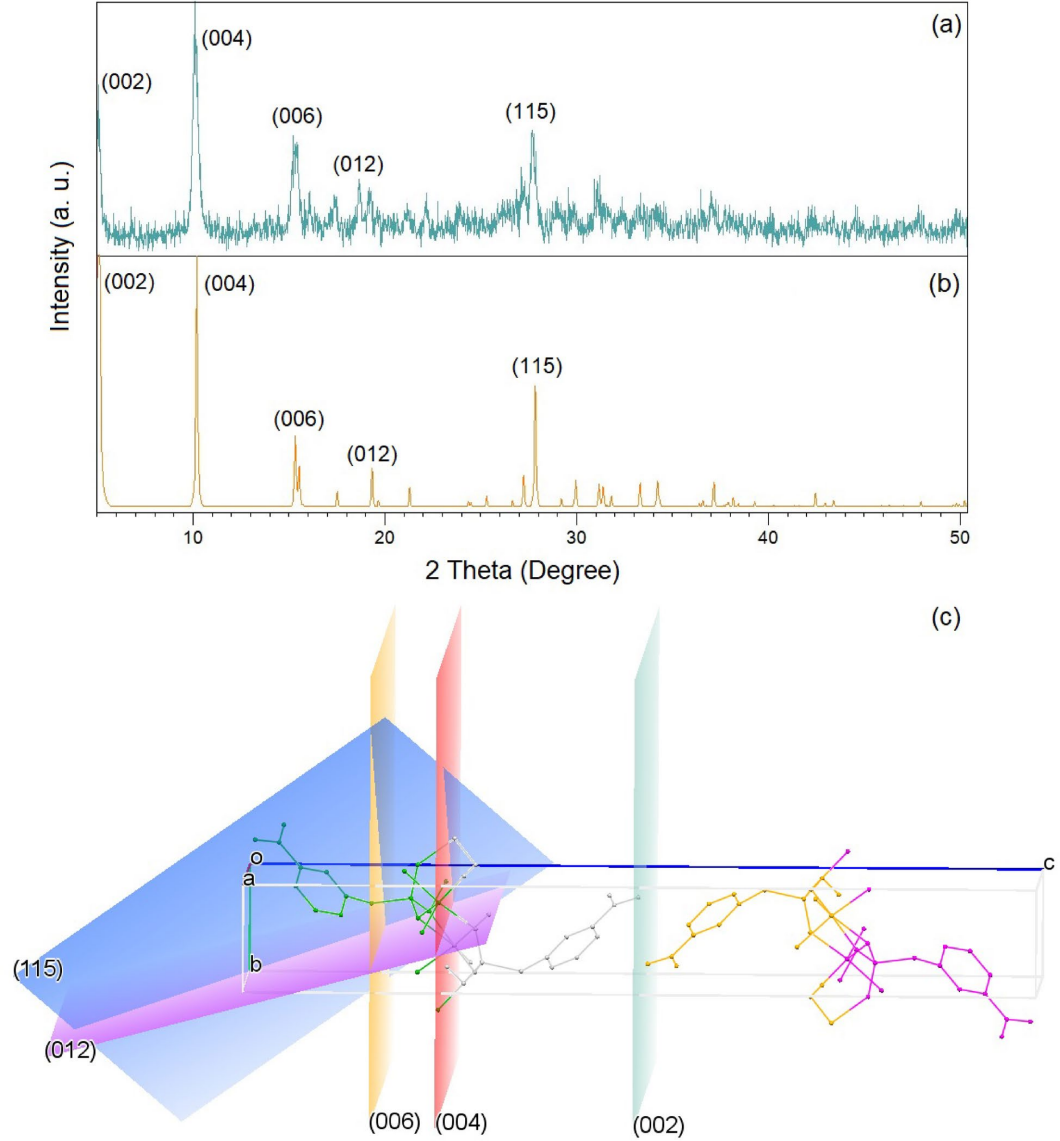
XRD patterns: (**a**) nanostructure Cu-MPB′, (**b**) single crystal Cu-MPB, and (**c**) simulated lattice planes in the crystal.

The experimental XRD pattern of Cu-MPB′, produced using the sonochemical method, is shown in Fig. 4a. Figure 4b presents the simulated XRD pattern of the same compound derived from single-crystal X-ray data of Cu-MPB. The simulated and experimental patterns demonstrated satisfactory agreement, with only slight variations in the 2*θ* values. This suggests that nanoparticles obtained via sonochemical synthesis Cu-MPB′ are identical to those produced via the solvothermal method. The divergence between the powder X-ray diffraction pattern and those derived from single-crystal X-ray analysis highlighted the broadening of peaks, suggesting that the particles were in the sub-micrometer dimension. The diffraction angles for both forms are 5.1°, 10.1°, and 15.1°, corresponding to the 2D crystallographic planes (002), (004), and (006), respectively. These planes confirm that Cu-MPB′ exhibits a nanosheet structure^41^. Figure 4c illustrates these crystallographic planes, with distances of 8.65 Å between (002) and (004) and 2.88 Å between (004) and (006). The diffraction angles of 19.1° and 28° correspond to planes (012) and (115), with the angle between them measuring 38.9°.

**Fig. 5:**
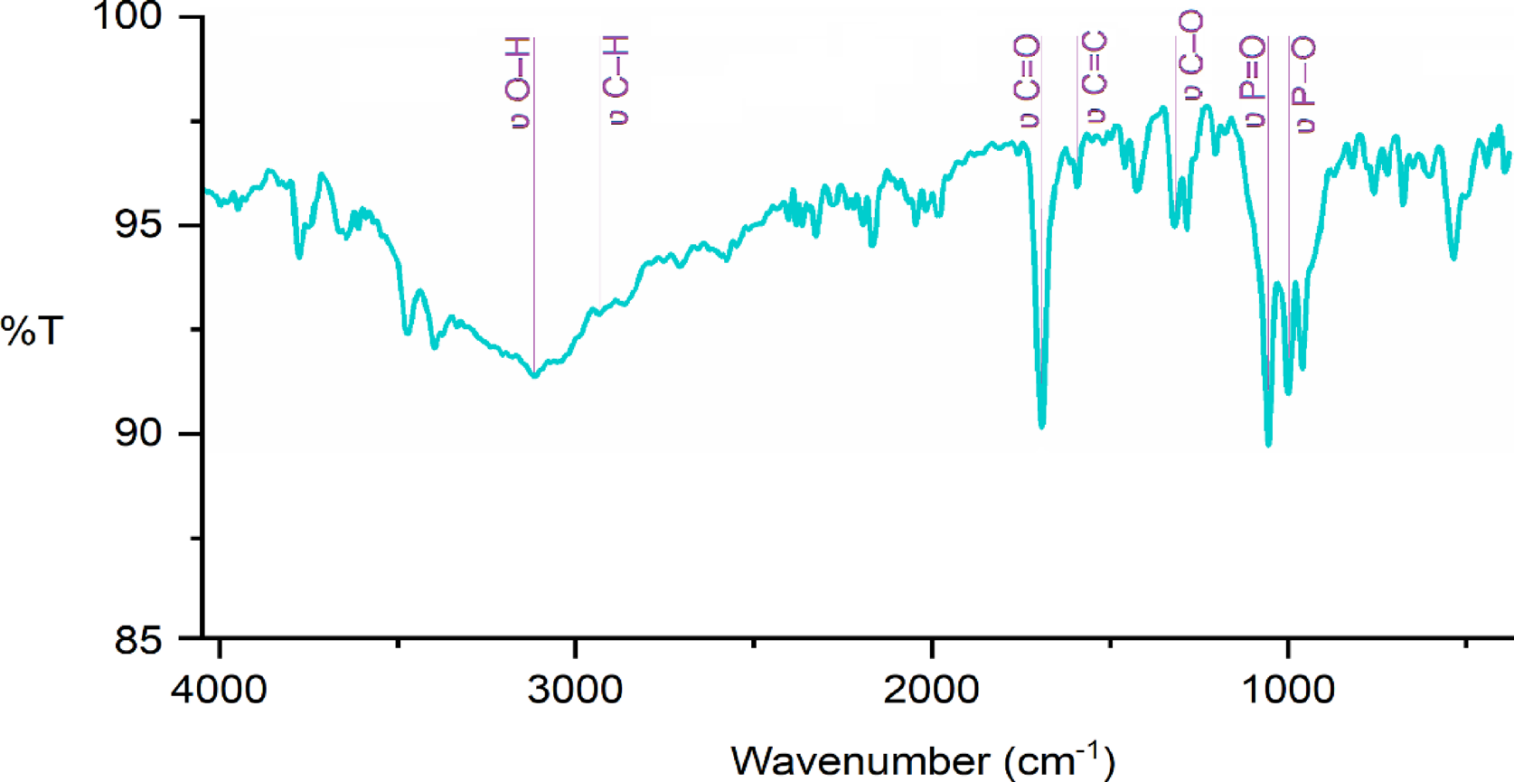
FT-IR spectrum of Cu-MPB′.

The FT-IR spectrum of Cu-MPB′ shows the peaks in the range of 2910–3250 cm^−1^ corresponding to the O–H bond stretching vibrations (Fig. 5). The signal at 2825 cm^−1^ indicates the vibrations of the C–H bonds in the aromatic groups. Furthermore, peaks at 1754 and 1249 cm^−1^, indicated carboxyl C = O and C–O stretching vibrations, respectively. Moreover, the signal at 1530 cm^−1^ indicates that C = C bonds are present in the aromatic structures. The presence of phosphate groups is confirmed by a high peak in the 1000–1100 cm^−1^ range, which shows the presence of P = O and P − O stretching vibrations^42^. Lastly, twisting modes of the aromatic ring and phosphate, together with bending vibrations of P − OH and C − H, are seen below 900 cm^−1^, which is in line with observations in the literature^43^.

**Fig. 6:**
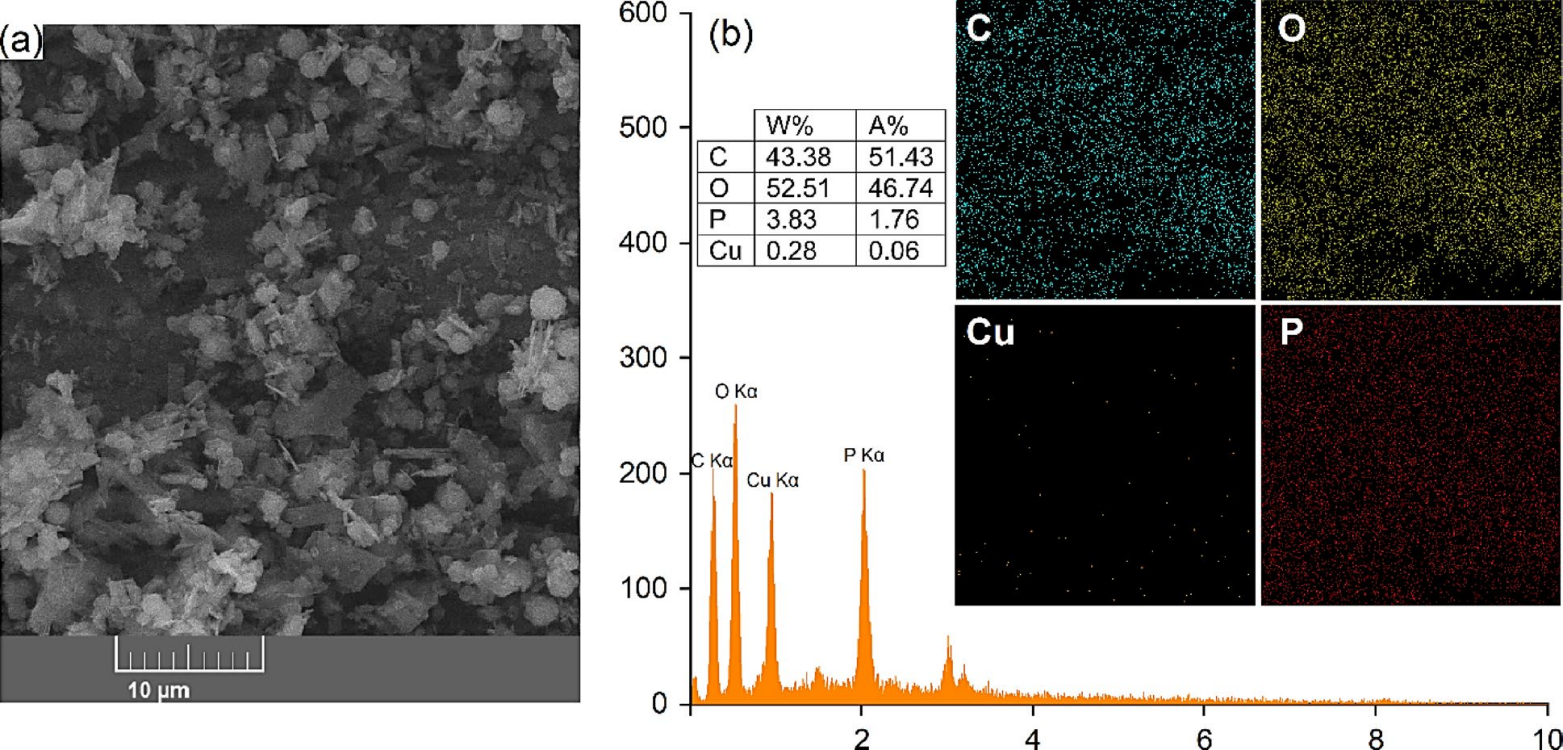
(**a**) SEM image, (**b**) EDX analysis and mapping of nanoparticles Cu-MPB′.

Scanning Electron Microscopy (SEM) images were utilized to analyze the micromorphological characteristics of Cu-MPB′. As illustrated in Fig. 6a, the Cu-MPB′ compound displayed uniformly spherical particles with sizes ranging from 100 nm to 5 μm. To assess the presence of anticipated elements within the material’s structure, energy dispersive X-ray (EDX) analysis was performed, yielding mapping spectra for Cu-MPB′. The EDX spectrum confirmed the presence of phosphorus (P), oxygen (O), copper (Cu), and carbon (C) in the sample, thereby validating the successful synthesis of Cu-MPB′ (Fig. 6b). Additionally, elemental mapping indicated a well-distributed, uniform, and homogeneous presence of Cu-MPB′ throughout the sample.

**Fig. 7:**
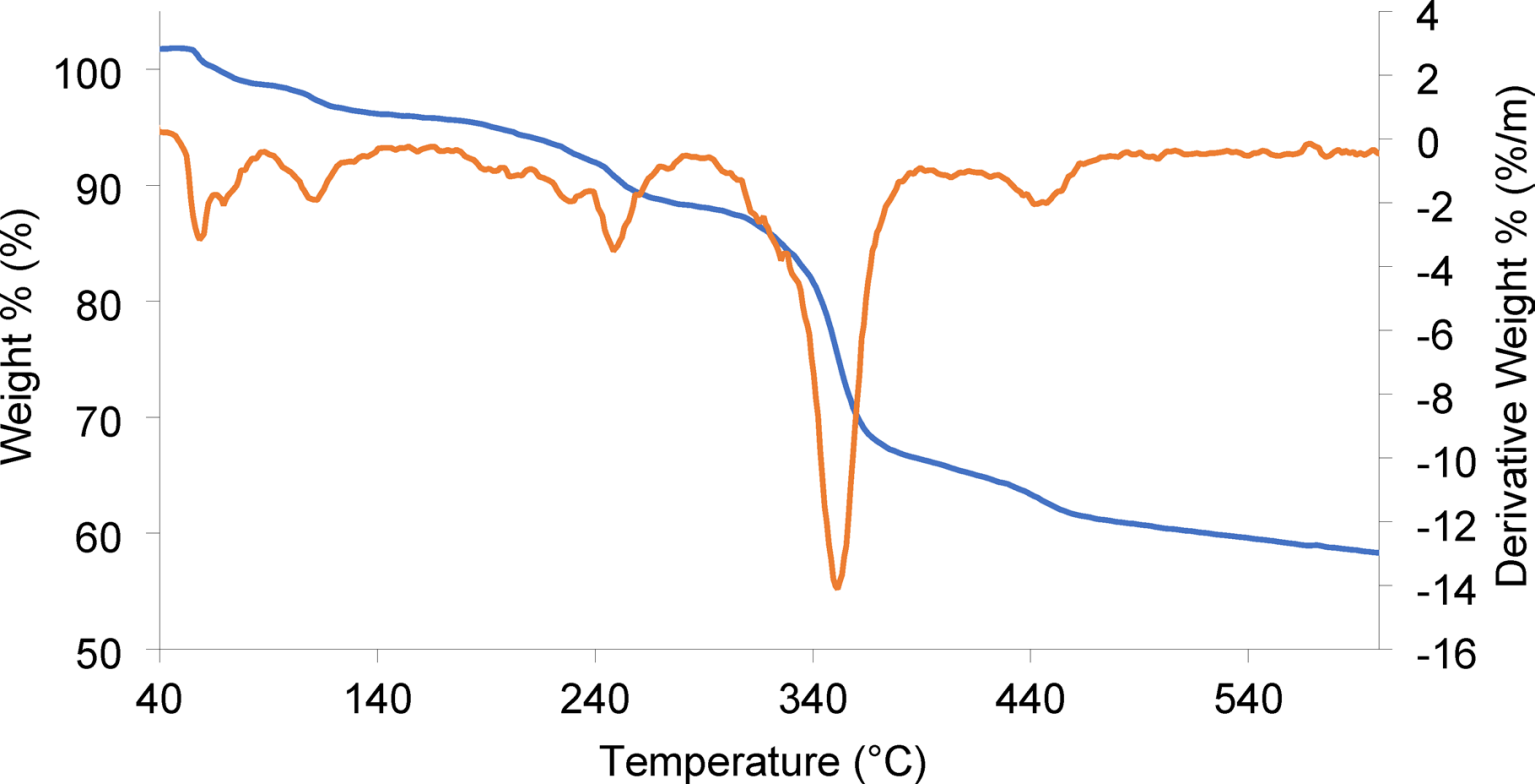
TGA/DTA diagram of Cu-MPB′.

TGA/DTA analysis of Cu-MPB′ was performed to investigate the stability and thermal degradation characteristics of the material (Fig. 7). An initial weight loss of 5.0% was recorded in the temperature range of 50 to 150 °C, which was ascribed to the elimination of non-coordinated water molecules^44^. A significant weight loss of 6.0% occurs between 150 and 250 °C, attributed to the release of coordinated water component. Between 250 and 350 °C, a more significant weight loss 22% was observed, indicating the decomposition of carboxylic groups of the benzoic acid and one oxygen atom of the phosphate component. Between 350 and 500 °C, the compound stabilizes at approximately 60% weight retention, indicating the formation of the residue (C₇H₇CuO₃P). Ultimately, above 500 °C, the TGA curve shows a gradual weight loss, which is attributed to decomposition of the organic ligand and the formation of copper oxides, which are relatively stable at higher temperatures^45,46^. This data underscores the thermal stability and degradation mechanisms relevant to the compound, providing insights into its thermal properties and potential applications in thermally sensitive environments.

**Fig. 8:**
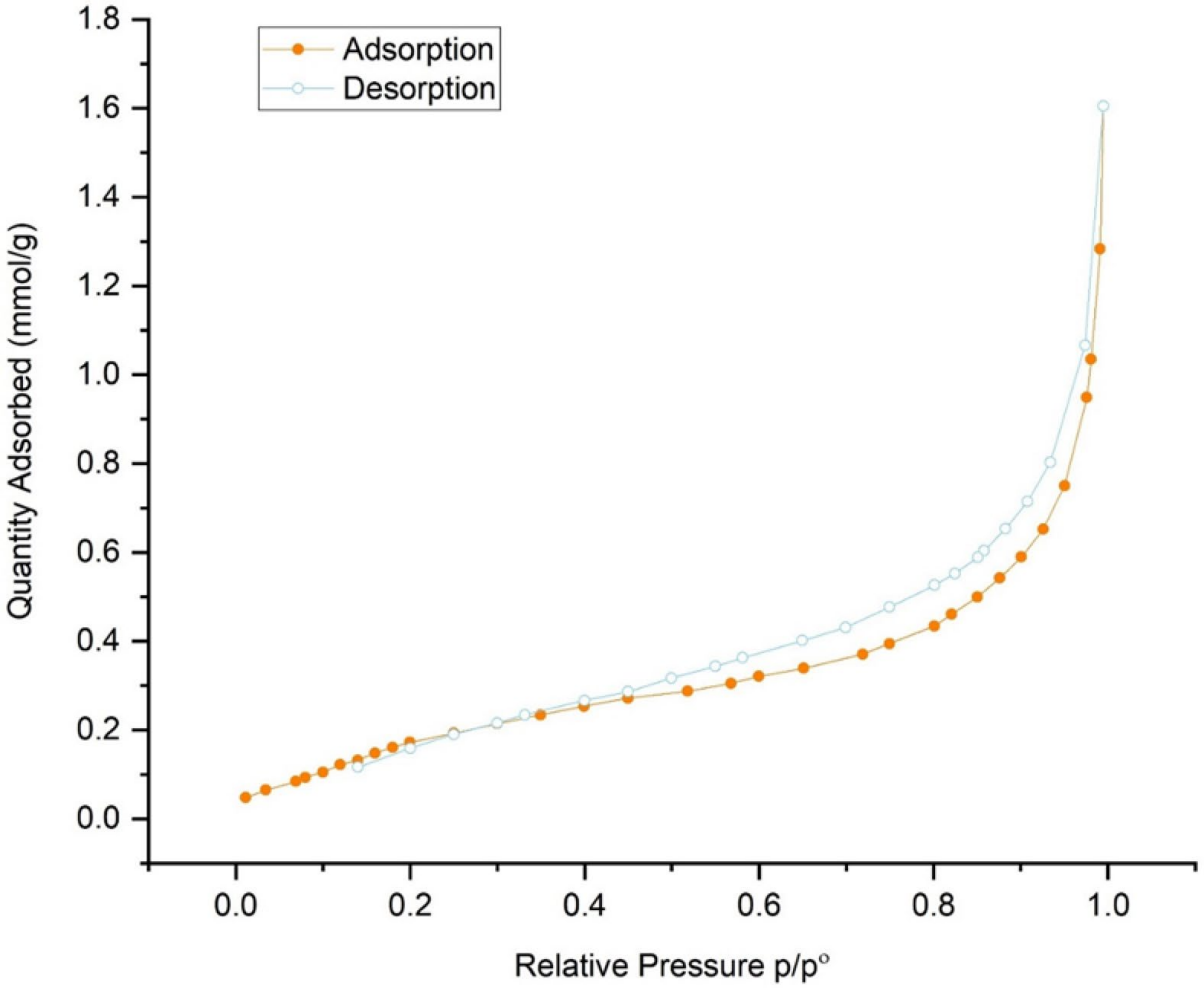
Nitrogen adsorption–desorption of compound Cu-MPB′.

According to the IUPAC classification^47^, compound Cu-MPB′ exhibited a type II curve with a prominent H3 hysteresis loop in its nitrogen adsorption/desorption isotherms. The calculations revealed that the material had a BET specific surface area of approximately 19.9 m^2^/g and a total pore volume of 0.06 cm^3^/g (Fig. 8). Furthermore, the Barrett-Joyner-Halenda (BJH) pore size distribution analysis indicated a mesoporous structure, with an average pore diameter of approximately 12.9 nm.

### Catalytic studies of Cu-MPB′ in the synthesis of tetrahydrobenzo[b]pyrans

The optimal conditions were investigated by assessing various parameters, including the choice of solvent, amount of the catalyst, temperature, and reaction duration (Table 2, entries 1–16). In the absence of nanocatalyst Cu-MPB′, no product was obtained after 120 min, indicating the importance of the presence of a catalyst in this reaction (Table 2, entries 1 and 2). Next, the effects of various solvents and reaction times were investigated. Interestingly, a moderate yield was obtained in EtOH, toluene, solvent-free, and H_2_O (Table 2, entries 3–6). Notably, the ratio of the water-ethanol mixtures had a significant effect on the reaction progress (Table 2, entries 7 and 8). Subsequently, the effect of increasing the catalyst loading was investigated. Importantly, the conversion increased with increasing catalyst loading, and the best result was observed using the catalyst (0.06 g) (Table 2, entries 11–14). The effect of increasing temperature on the reaction efficiency was also investigated (Table 2, entries 15 and 16).

**Table 2:** The effect of solvent, time, temperature, and catalyst loading in the synthesis of tetrahydrobenzo[b]pyran.


**Entry**	**Catalyst (g)**	**Solvent**	**Time (min)**	**T (°C)**	**Yield (%)**
1	-	H_2_O	120	35	No reaction
2	-	EtOH	120	35	No reaction
3	(0.06)	H_2_O	60	35	70
4	(0.06)	EtOH	60	35	55
5	(0.06)	Toluene	60	35	25
6	(0.06)	Solvent-free	60	35	34
7	(0.06)	H_2_O/EtOH(1:1)	60	35	95
8	(0.06)	H_2_O/EtOH(1:2)	60	35	95
9	(0.06)	H_2_O/EtOH(1:1)	40	35	95
10	(0.06)	H_2_O/EtOH(1:1)	30	35	95
11	(0.06)	H_2_O/EtOH(1:1)	25	35	95
12	(0.04)	H_2_O/EtOH(1:1)	25	35	82
13	(0.02)	H_2_O/EtOH(1:1)	25	35	75
14	(0.1)	H_2_O/EtOH(1:1)	25	35	95
15	(0.06)	H_2_O/EtOH(1:1)	25	60	84
16	(0.06)	H_2_O/EtOH(1:1)	25	80	79

As shown in Table 2, among the studied solvents, the water/ethanol mixture (1:1) demonstrates the best performance and provides the highest yield compared to water, ethanol, toluene, and solvent-free conditions. In this solvent, reducing the reaction time from 60 to 25 min results in a 95% yield, which is comparable to that achieved with longer reaction times. The optimal amount of catalyst is 0.06 g, as reducing it to 0.04 g–0.02 g decreases the yield, while increasing it to 0.1 g does not affect the yield. Therefore, the best conditions for synthesis involve using 0.06 g of Cu-MPB′ in a water/ethanol (1:1) solvent with a reaction time of 25 min at 35 °C, achieving a maximum yield of 95%.

According to optimal conditions, condensation of different benzaldehyde derivatives has been investigated, and the results of these studies are presented in Table 3. The benzaldehydes with electron-donating, electron-withdrawing, aliphatic, and hetero groups converted to relatively tetrahydrobenzo[b]pyran products at relatively short times with very good efficiencies (Table 3, entries 2–8). The benzaldehydes with electron-withdrawing groups (Table 3, entries 2, 3, and 5) were converted into tetrahydrobenzo[b]pyran products at shorter times and higher yields in comparison to the benzaldehyde, although the benzaldehydes with electron-donating groups converted at longer times and lower yields. Also, the benzaldehyde derivative with the *meta* position compared to the *para* position has a longer reaction time and less yield (Table 3, entries 2 and 5). The use of aliphatic and hetero aldehyde groups produced relatively less product (Table 3, entries 7 and 8). However, a good yield of products was achieved under mild conditions, which required only moderate temperature and green solvents. The reactions were carried out quickly, and the substrates had to be completely converted in a comparatively short time. This highlights the broad applicability of the catalyst in the synthesis of tetrahydrobenzo[b]pyran derivatives, demonstrating its versatility and potential for wider applications in organic synthesis. Some selected spectroscopic data of the synthesized products are shown in the Supporting Information Section (Figures S1-S14).

**Table 3:** Synthesis of tetrahydrobenzo[b]pyrans using 0.06 g of nanocatalyst Cu-MPB′ at 35 °C.


**Entry**	**Product**	**Time (min)**	**Yield (%)**	**Found M. P. (°C)**	**Reported M. P. (°C)**	**TON** ^**a**^	**TOF** ^**b**^	**Ref.** ^**c**^
1		25	95	223–225	226–228	4.75	11.58	^48^
2		15	96	176–178	177–179	4.8	19.2	^49^
3		20	94	213–215	215–217	4.7	14.2	^50^
4		30	90	200–202	196–198	4.5	9	^51^
5		20	95	186–189	189–190	4.75	14.3	^52^
6		35	85	217–219	214–215	4.25	7.3	^53^
7		45	83	211–214	213–215	4.15	5.5	^54^
8		40	80	177–180	174–178	4	6	^55^

^a^TON = turnover number (moles of desired product formed per mole of the catalyst), ^b^TOF = turnover frequency (turnover number per unit time). ^c^The reference of reported products.

**Fig. 9:**
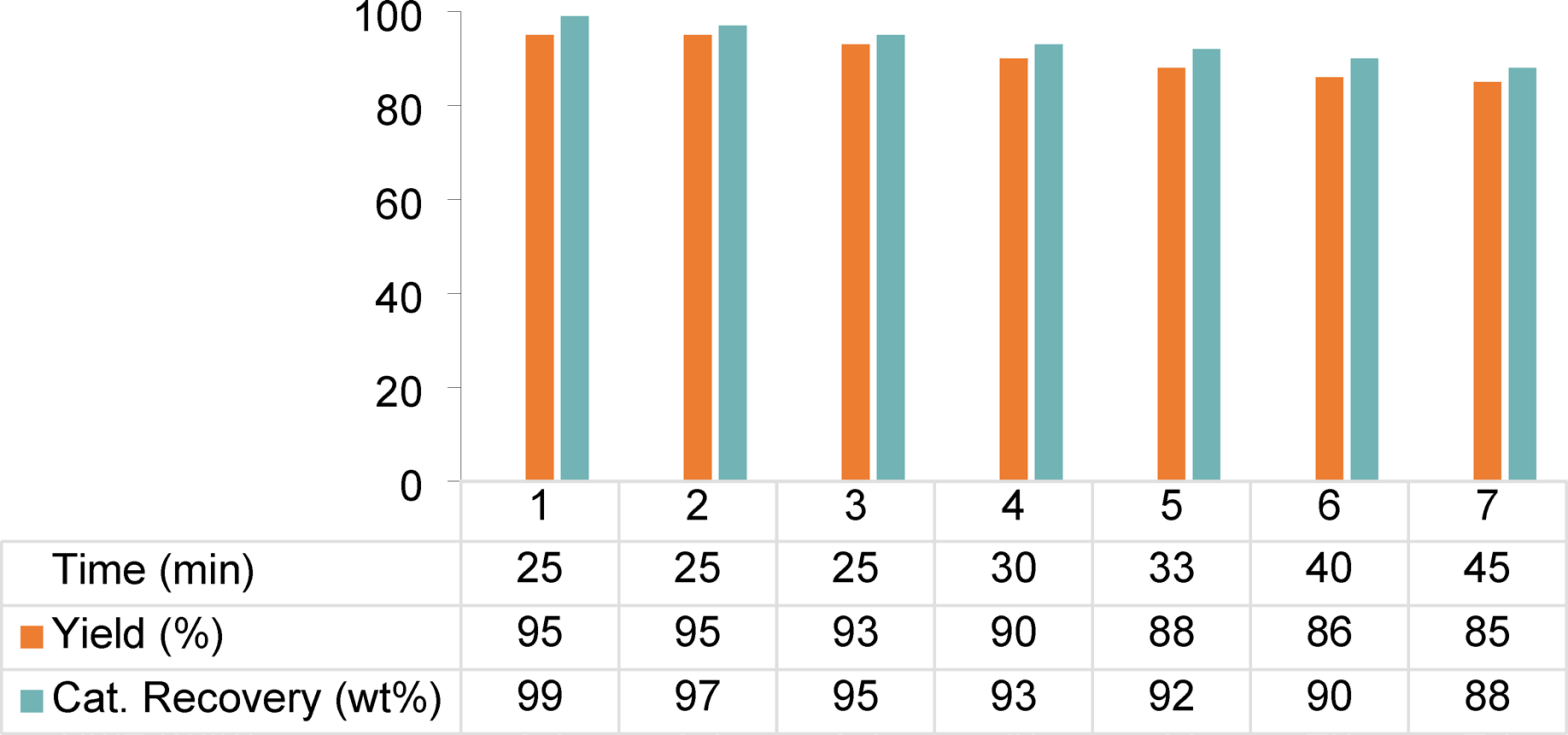
Recycling, reusability, and recovery of Cu-MPB′.

The remarkable catalytic efficiency inspired us to investigate the critical aspects of the longevity of MOF catalysts. The recoverability and reusability of the designed heterogeneous catalyst were investigated in the reaction under optimized conditions (Fig. 9). The heterogeneous catalyst was used with excellent yields up to seven times, with a slight reduction in the synthesis of tetrahydrobenzo[b]pyran. The recyclability results of catalyst Cu-MPB′ showed that the synthesis yield remained relatively constant (approximately 90 to 95%) in the initial cycles (1–4), but gradually decreased with repeated use, reaching 85% in the 7th cycle. This decrease can be attributed to the washing away of active sites or the accumulation of byproducts^56^. Therefore, it can be concluded that the catalyst should be regenerated after four uses to achieve maximum efficiency.

**Fig. 10:**
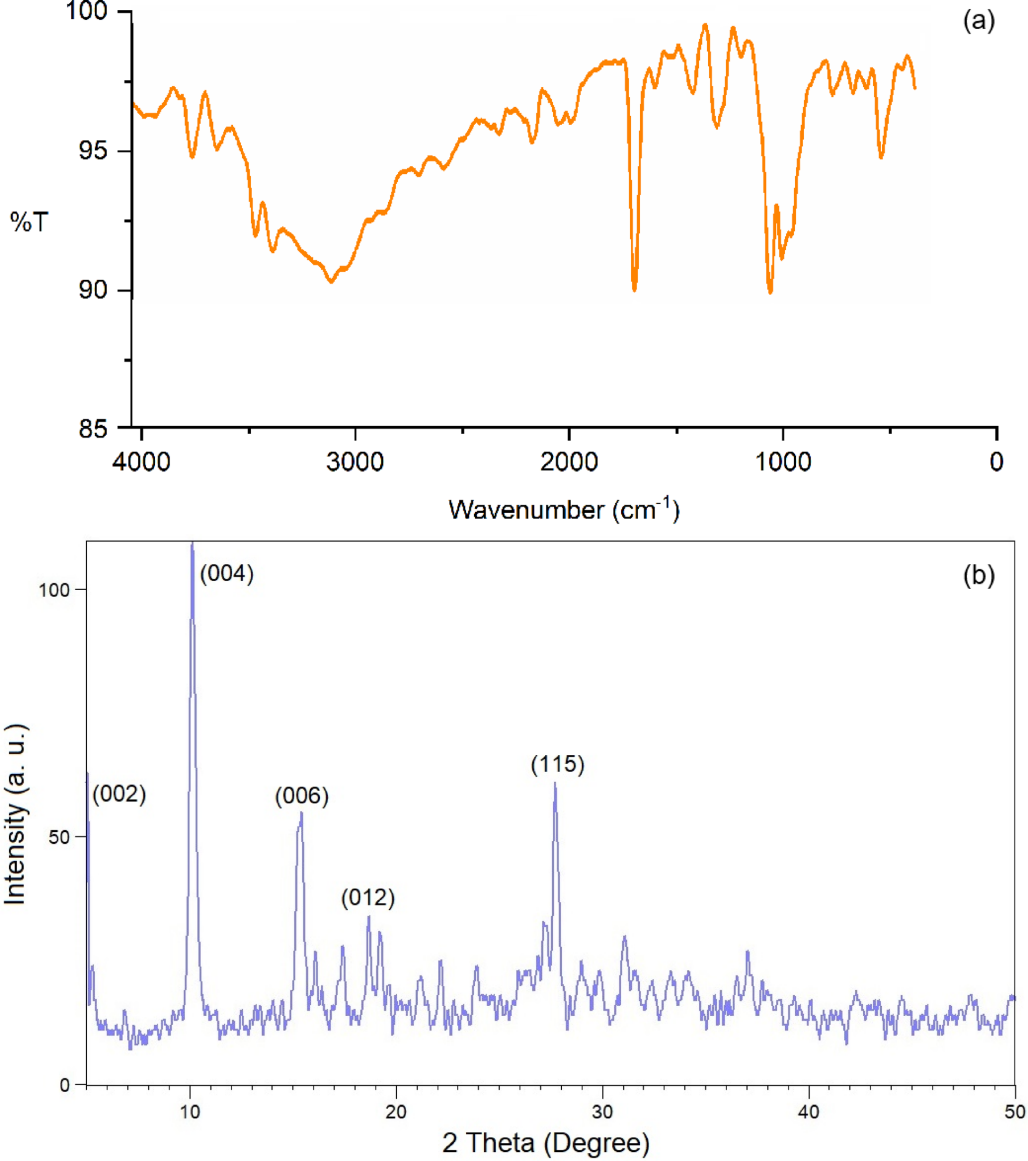
**(a)** FT-IR spectrum, and (**b)** PXRD pattern of recovered Cu-MPB′.

Next, to study the chemical and structural stability of the catalyst under operating conditions, FT-IR and PXRD analyses of the recovered catalyst were conducted after the seven-run. The FT-IR spectrum of the recycled catalyst exhibits a high level of consistency and overlap with the spectrum of the fresh catalyst, confirming that the structural integrity of the catalyst remains well-preserved following the recycling process (Fig. 10a).

The PXRD of the recovered Cu-MPB′ also illustrated five peaks at 2*θ* of 5.1°, 10.1°, 15.1°, 19.3°, and 27.8°, which are in good agreement with the PXRD pattern of the fresh nanocatalyst, proving the high stability of the crystalline structure of Cu-MPB′ nanoparticles during the reaction process (Fig. 10b).

Finally, to compare efficiency and to demonstrate the importance of the proposed method, some of the reported cases in various literature have been gathered in Table 4. Nanocatalyst Cu-MPB′, with its simple and rapid single-step synthesis, exhibits high recyclability and efficiently facilitates the synthetic reaction of tetrahydrobenzo[b]pyran in green solvents. By achieving high efficiency at optimal temperature and time, this catalyst offers significant potential for advancing sustainable practices in organic synthesis.

**Table 4:** The comparison of the conditions and the results of reported works with Cu-MPB′.

Catalyst	Conditions	Time (min)	Yield (%)	Ref.
HMS/Pr-Rh-Zr	PEG, 80 °C	30	87	^57^
Fe_3_-xTixO_4_@SO_3_HNPs	H_2_O/EtOH (1:1), reflux	60	95	^58^
2-aminopyridine	EtOH, reflux	8	91	^59^
GO@[PBVIm]HSO_4_	H_2_O/EtOH (1:1), 80 °C	30	92	^60^
H_2_PO_4_-SCMNPs	Solvent-free, 80 °C	20	92	^24^
GO-ANSA	EtOH, reflux	30	94	^61^
Fe_3_O_4_@SiO_2_-NH_2_/GO/IL-Mn	H_2_O, rt	40	95	^62^
CaO@SiO_2_-SO_3_H	H_2_O, 50 °C	20	93	^63^
**Cu-MPB′**	**H**_**2**_**O/EtOH (1:1)**,** 35 °C**	**25**	**95**	**This Work**

**Fig. 11:**
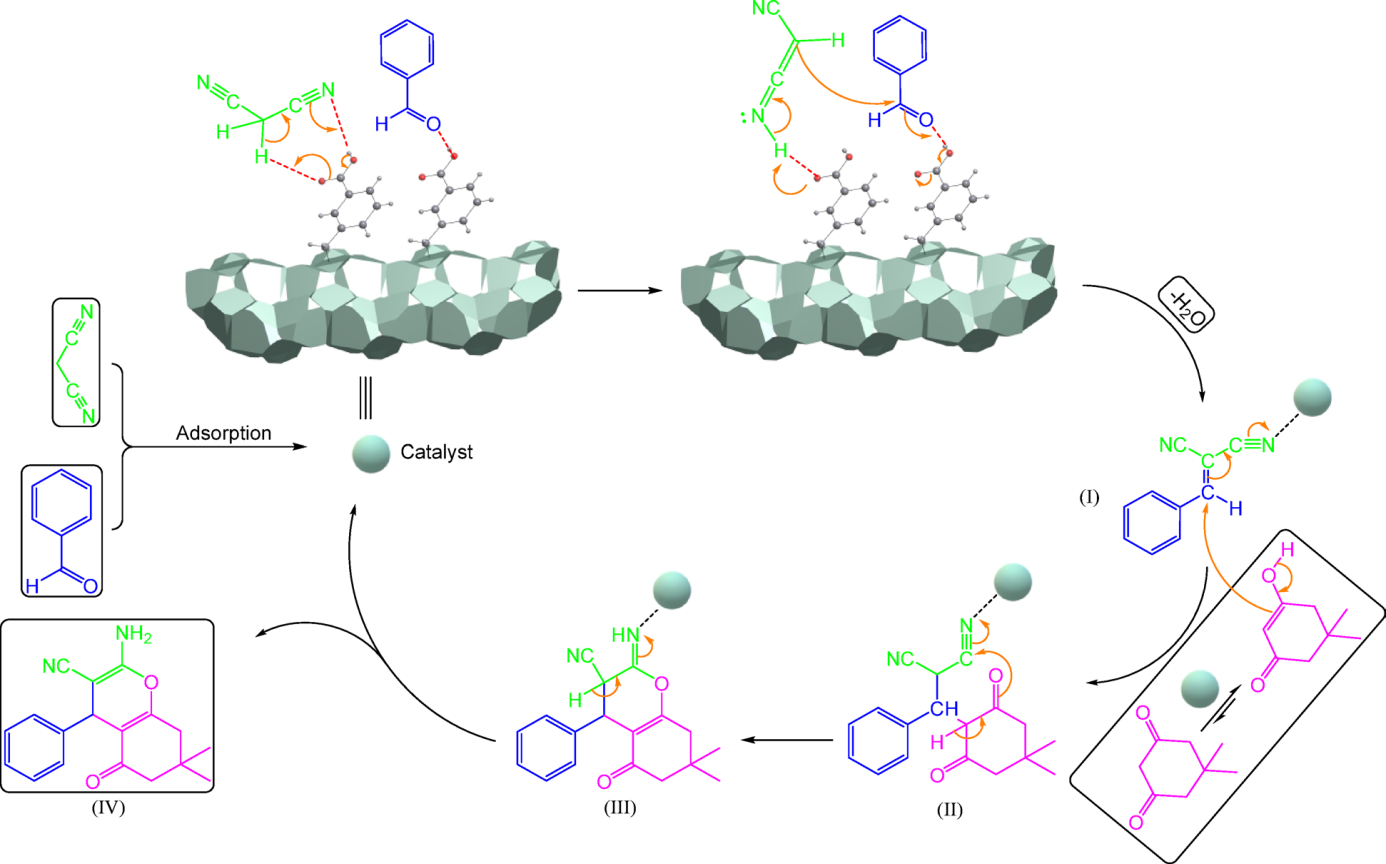
The proposed mechanism for the synthesis of tetrahydrobenzo[b]pyrans using the nanocatalyst Cu-MPB′.

Figure 11 depicts a feasible pathway for tetrahydrobenzo[b]pyran synthesis using catalyst Cu-MPB′. The process begins with the adsorption of aldehyde and malononitrile compounds by hydrogen binding on the carboxyl group of catalyst Cu-MPB′, which amplifies the electrophilic properties of both the aldehyde’s carbonyl group and malononitrile. This is followed by a Knoevenagel condensation reaction between activated malononitrile and activated aldehyde. The subsequent dehydration step leads to the formation of the 2-benzylidenemalononitrile intermediate (**I**). In continuous, the addition of enolizable dimedone to the 2-benzylidenemalononitrile intermediate (**II**), followed by continuous intramolecular cyclization, gives intermediate (**III**). Finally, tautomerization yielded the corresponding tetrahydrobenzo[b]pyran (**IV**)^64^.

## Conclusion

This study successfully synthesized a novel two-dimensional Cu-MOF, Cu-MPB′, via an ultrasonic method and demonstrated its efficacy as a highly active, reusable heterogeneous catalyst for the green synthesis of tetrahydrobenzo[b]pyrans. Cu-MPB′ exhibited excellent catalytic performance under mild, aqueous ethanol conditions, achieving high yields with low catalyst loading (0.06 g) and short reaction times (as low as 25 min) across a broad substrate scope. Critically, the catalyst retained over 85% yield after seven reuse cycles, confirming its robust recyclability. Comparative analysis established Cu-MPB′’s superiority in efficiency, operational simplicity, and environmental compatibility over existing systems. This work contributes a new member to the 2D MOF family and validates sonochemical synthesis as a scalable, sustainable route for producing high-performance MOF nanocatalysts.

## Supplementary Information

Below is the link to the electronic supplementary material.

Supplementary Material 1

## Data Availability

The datasets generated during and/or analyzed during the current study are available from the corresponding author on reasonable request.
