# Supplementary material for: Design and catalytic performance of a novel two-dimensional copper metal-organic framework for green synthesis of tetrahydrobenzo[b]pyrans
Source: Sci Rep. 2025 Oct 3;15:34495. doi: 10.1038/s41598-025-14653-1 (PMC12494978; doi:10.1038/s41598-025-14653-1)
Supplement: Supplementary file 1 — Supplementary Material 1 [file 41598_2025_14653_MOESM1_ESM.docx]

**Supporting Information**

**Design and catalytic performance of a novel two-dimensional copper metal-organic framework for green synthesis of tetrahydrobenzo[b]pyrans**

Ehsan Joukar Bahaderani^a^, Khosro Mohammadi^*a^, Payam Hayati^*b^, Jan Janczak^c^

*^a^ Chemistry Department, Faculty of Nano and Bio Sciences and Technology, Persian Gulf University, Bushehr 75169, Iran*

*^b^ Department of Biotechnology and Life Sciences, University of Insubria, Via Jean Henry Dunant, 3, 21100 Varese, Italy*

*^c^ Institute of Low Temperature and Structure Research, Polish Academy of Sciences, P. O. Box 1410, Okólna 2 Str., 50-422 Wroclaw, Poland*

Email: [khmohammadi@pgu.ac.ir](mailto:khmohammadi@pgu.ac.ir); [khmohammadi@yahoo.com](mailto:khmohammadi@yahoo.com), payamhayati@yahoo.com, payam.hayati@uninsubria.it

**Table S1**. Crystallographic data for Cu-MPB.

| **Compound** | **[Cu(H_2_O)(*m*-PO_3_CH_2_C_6_H_4_CO_2_H)]_n_ (**Cu-MPB**)** |
| --- | --- |
| Chemical formula | C_8_H_9_CuO_6_P |
| *M*_r_ | 295.66 |
| Crystal system, space group | Monoclinic, *P*2_1_/*n* |
| Temperature (K) | 295(2) |
| *a*, *b*, *c* (Å) | 5.7362 (5), 4.7509 (4), 34.635 (3) |
| *β* (°) | 92.327 (7) |
| *V* (Å^3^) | 943.09 (14) |
| *Z* | 4 |
| *D*_calc_ (g·cm^–3^) | 2.082 |
| Radiation type, wavelength, λ (Å) | Mo *Kα* , 0.71073 |
| *µ* (mm^−1^) | 2.50 |
| *F*(000) | 596 |
| Crystal size (mm) | 0.24 × 0.20 × 0.15 |
| *T*min, *T*max | 0.794, 1.000 |
| *θ* range(°) | 3.5-27.4 |
| Absorption correction | multi-scan |
| Reflections collected / unique / observed | 26181, 2167, 1913 |
| *R*int | 0.064 |
| Refinement on | *F*^2^ |
| *R*[*F*^2^ > 2σ(*F*^2^)] | 0.0745 |
| *wR*(*F*^2^ all reflections) | 0.1655 |
| Goodness-of-fit, *S* | 1.001 |
| Δ*ρ*_max_, Δ*ρ*_min_ (e Å^−3^) | +1.442, -0.844 |

*wR*={Σ [*w*(*F*_o_^2^–*F*_c_^2^)^2^]/Σ*wF*_o_^4^}^½^; *w* = 1/[*σ*^2^(*F*_o_^2^) + (0.0549*P*)^2^ + 16.5039*P*] where *P* = (*F*_o_^2^ + 2*F*_c_^2^)/3

**The experimental data of tetrahydrobenzo[b]pyrans**

*2-amino-7,7-dimethyl-5-oxo-4-phenyl-5,6,7,8-tetrahydro-4H-chromene-3-carbonitrile (****1****)*

White solid; Yield: 95%; TLC: ethyl acetate/hexane (1:3); Melting point: 223-225; IR (KBr, cm^−1^): 3394, 3324 (NH_2_, stretching vibration), 3208 (=C–H, stretching vibration sp^2^), 2965 (C–H, stretching vibration sp^3^), 2198 (CN, stretching vibration), 1666 (C=O, stretching vibration), 1457, 1411 (C=C, Ar stretching vibration sp^2^), 1214 (C–O, stretching vibration).

*2-amino-7,7-dimethyl-4-(4-nitrophenyl)-5-oxo-5,6,7,8-tetrahydro-4H-chromene-3-carbonitrile (****2****)*

White solid; Yield: 96%; TLC: ethyl acetate/hexane (1:4); Melting point: 176-178; IR (KBr, cm^−1^): 3359, 3316 (NH_2_, stretching vibration), 3181 (=C–H, stretching vibration sp^2^), 2931 (C–H, stretching vibration sp^3^), 2194 (CN, stretching vibration), 1662 (C=O, stretching vibration), 1519, 1357 (NO_2_ stretching vibration), 1469, 1407 (C=C, Ar stretching vibration sp^2^), 1222 (C–O, stretching vibration).

*2-Amino-4-(4-chlorophenyl)-7,7-dimethyl-5-oxo-6,6,8,8-tetrahydro-4H-chromene-3-carbonitrile (****3****)*

White solid; Yield: 94%; TLC: ethyl acetate/hexane (1:4); Melting point: 213-215; IR (KBr, cm^−1^): 3378, 3320 (NH_2_, stretching vibration), 3181 (=C–H, stretching vibration sp^2^), 2958 (C–H, stretching vibration sp^3^), 2185 (CN, stretching vibration), 1673 (C=O, stretching vibration), 1488, 1403 (C=C, Ar stretching vibration sp^2^), 1214 (C–O, stretching vibration); ^1^H NMR (300 MHz, DMSO-d_6_): *δ* (ppm) 1.05 (s, 3H), 1.08 (s, 3H), 2.09 (d, J = 16.2 Hz, 1H), 2.30 (d, J=16.2 Hz, 1H), 2.49 (s, 2H), 4.17 (s, 1H), 7.06 (s, 2H), 7.11 (d, J = 6.6 Hz, 2H), 7.50 (d, J = 6.6 Hz, 2H); ^13^C NMR (75 MHz, DMSO-d_6_): *δ* (ppm) 20.9, 26.9, 28.9, 31.9, 35.4, 41.2, 50.3, 58.4, 79.8, 112.6, 120.2, 130.5, 131.8, 144.5, 158.6, 163.2, 196.0.

*2-amino-4-(4-methoxyphenyl)-7,7-dimethyl-5-oxo-5,6,7,8-tetrahydro-4H-chromene-3-carbonitrile (****4****)*

White solid; Yield: 90%; TLC: ethyl acetate/hexane (1:3); Melting point: 200-202; IR (KBr, cm^−1^): 3355, 3304 (NH_2_, stretching vibration), 3185 (=C–H, stretching vibration sp^2^), 2958 (C–H, stretching vibration sp^3^), 2190 (CN, stretching vibration), 1654 (C=O, stretching vibration), 1461, 1415 (C=C, Ar stretching vibration sp^2^), 1251 (C–O, stretching vibration).

*2-Amino-4-(3-nitrophenyl)-7,7-dimethyl-5-oxo-6,6,8,8-tetrahydro-4H-chromene-3-carbonitrile (****5****)*

White solid; Yield: 95%; TLC: ethyl acetate/hexane (1:4); Melting point: 186-189; IR (KBr, cm^−1^): 3432, 3332 (NH_2_, stretching vibration), 3295 (=C–H, stretching vibration sp^2^), 2958 (C–H, stretching vibration sp^3^), 2190 (CN, stretching vibration), 1666 (C=O, stretching vibration), 1527, 1353 (NO_2_ stretching vibration), 1469, 1319 (C=C, Ar stretching vibration sp^2^), 1211 (C–O, stretching vibration); ^1^H NMR (300 MHz, DMSO-d_6_): *δ* (ppm) 0.98 (s, 3H), 1.06 (s, 3H), 2.13 (d, J = 15.9 Hz, 1H), 2.29 (d, J = 15.9 Hz, 1H), 2.52 (s, 2H), 4.44 (s, 1H), 7.22 (s, 2H), 7.67 (m, 2H), 8.00 (s, 1H), 8.10 (d, J = 7.5 Hz, 1H); ^13^C NMR (75 MHz, DMSO-d_6_): *δ* (ppm): 21.1, 27.4, 28.6, 32.4, 35.5, 40.6, 50.2, 57.5, 111.9, 120.9, 122.2, 122.4, 130.2, 134.5, 147.4, 148.1, 159.4, 163.9, 196.3.

*2-Amino-4-(4-methylyphenyl)-7,7-dimethyl-5-oxo-6,6,8,8-tetrahydro-4H-chromene-3-carbonitrile (****6****)*

White solid; Yield: 85%; TLC: ethyl acetate/hexane (1:3); Melting point: 217–219; IR (KBr, cm^−1^): 3424, 3328 (NH_2_, stretching vibration), 3193 (=C–H, stretching vibration sp^2^), 2923 (C–H, stretching vibration sp^3^), 2190 (CN, stretching vibration), 1673 (C=O, stretching vibration), 1461, 1407 (C=C, Ar stretching vibration sp^2^), 1211 (C–O, stretching vibration); ^1^H NMR (300 MHz, DMSO-d_6_): *δ* (ppm) 1.10 (s, 3H), 1.16 (s, 3H), 2.12 (d, J = 15.7 Hz, 1H), 2.22 (d, J = 15.7 Hz, 1H), 2.30 (s, 3H), 2.54 (s, 2H), 4.40 (s, 1H), 7.02 (s, 2H), 7.16-7.33 (m, 4H); ^13^C-NMR (75 MHz, DMSO-d_6_): *δ* (ppm) 21.3, 27.7, 29.0, 32.5, 35.3, 40.9, 50.7, 56.8, 63.8, 113.9, 118.6, 127.8, 129.3, 136.8, 140.1, 157.2, 159.6, 161.8, 196.1.

*2-amino-7,7-dimethyl-5-oxo-4-(thiophen-2-yl)-5,6,7,8-tetrahydro-4H-chromene-3-carbonitrile (****7****)*

White solid; Yield: 83%; TLC: ethyl acetate/hexane (1:3); Melting point: 211–214; IR (KBr, cm^−1^): 3432, 3332 (NH_2_, stretching vibration), 3205 (=C–H, stretching vibration sp^2^), 2958 (C–H, stretching vibration sp^3^), 2190 (CN, stretching vibration), 1666 (C=O, stretching vibration), 1469, 1419 (C=C, Ar stretching vibration sp^2^), 1211 (C–O, stretching vibration).

*2-amino-7,7-dimethyl-5-oxo-4-propyl-5,6,7,8-tetrahydro-4H-chromene-3-carbonitrile (****8****)*

White solid; Yield: 80%; TLC: ethyl acetate/hexane (1:2); Melting point: 177-180; IR (KBr, cm^−1^): 3378, 3320 (NH_2_, stretching vibration), 2958 (C–H, stretching vibration sp^3^), 2186 (CN, stretching vibration), 1673 (C=O, stretching vibration), 1214 (C–O, stretching vibration).


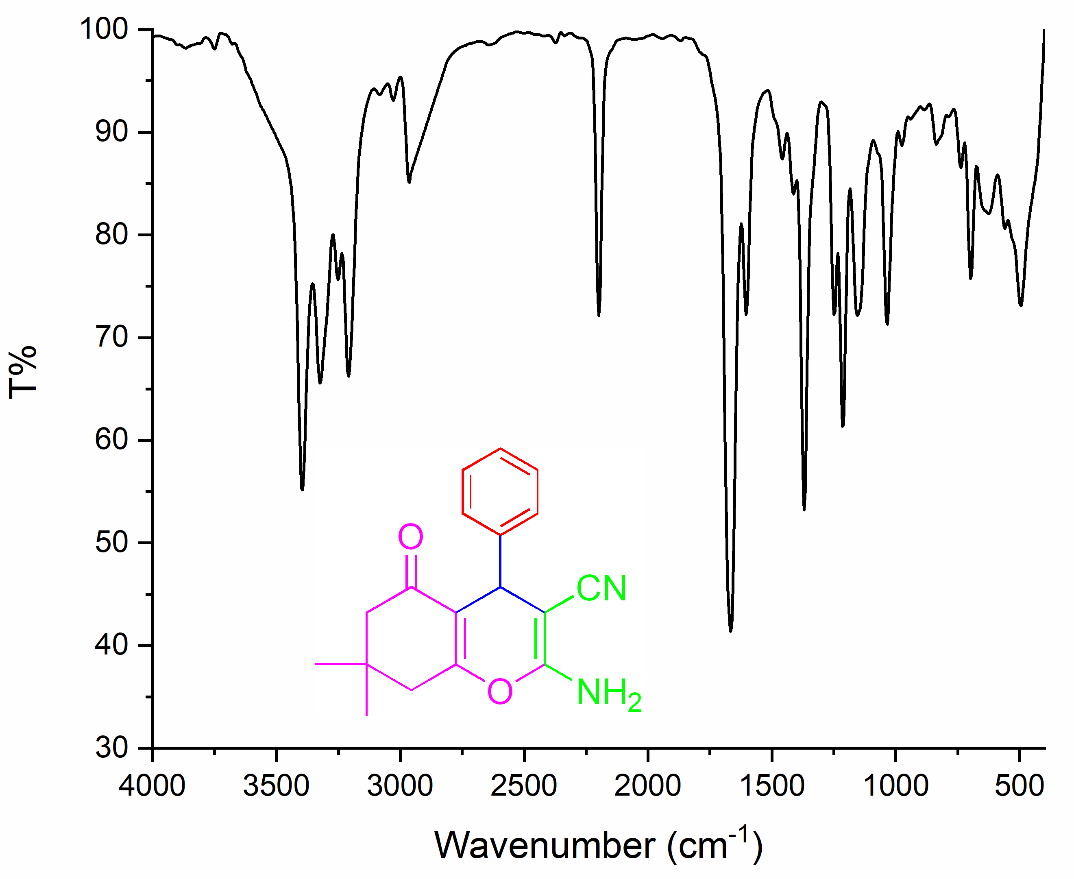


**Figure S1.** FT-IR spectra of compound **1**.


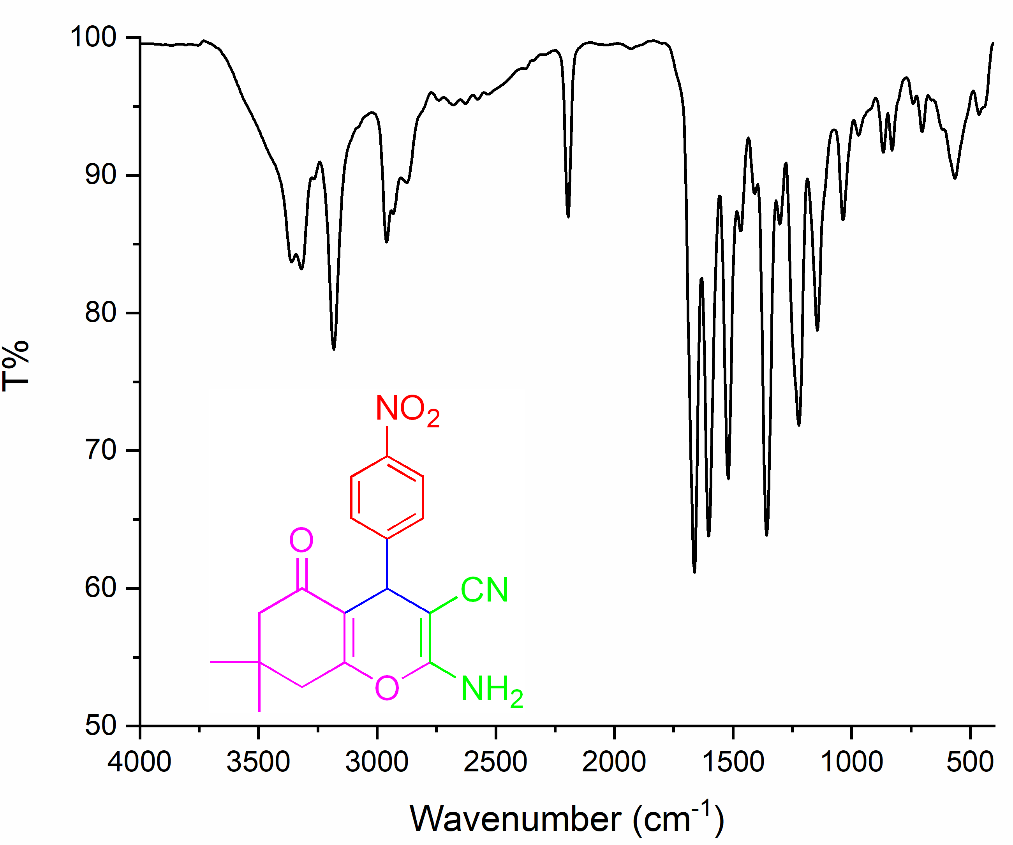


**Figure S2.** FT-IR spectra of compound **2**.


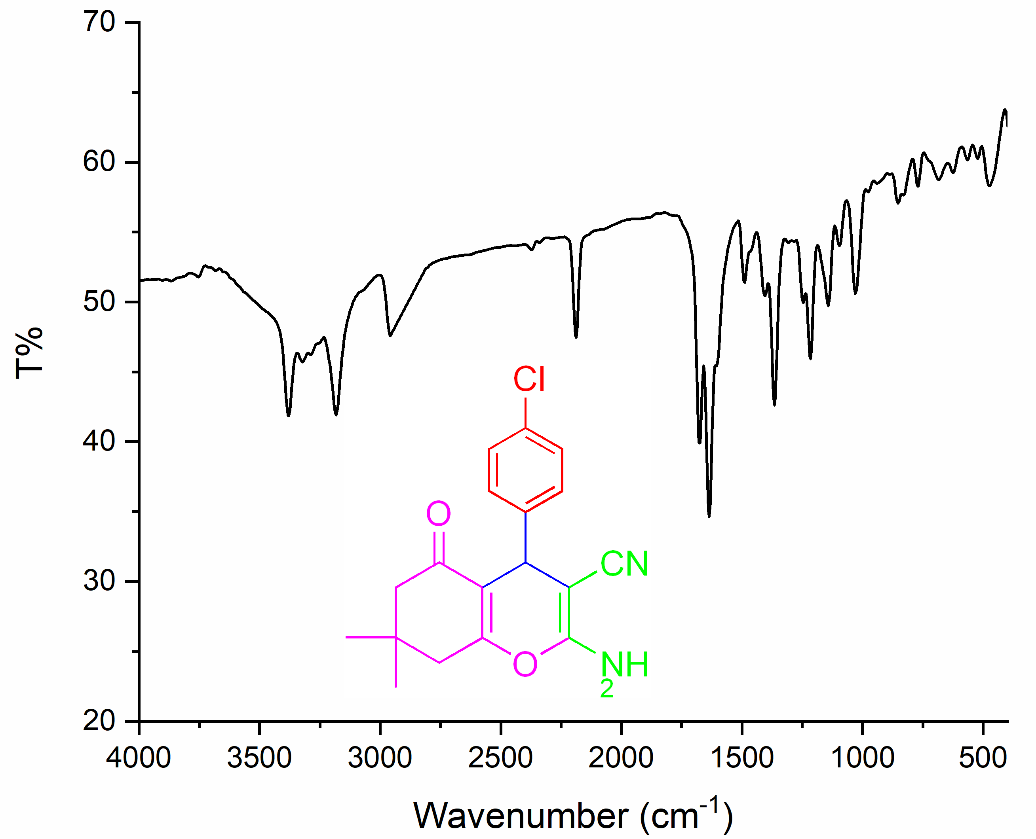


**Figure S3.** FT-IR spectra of compound **3**.


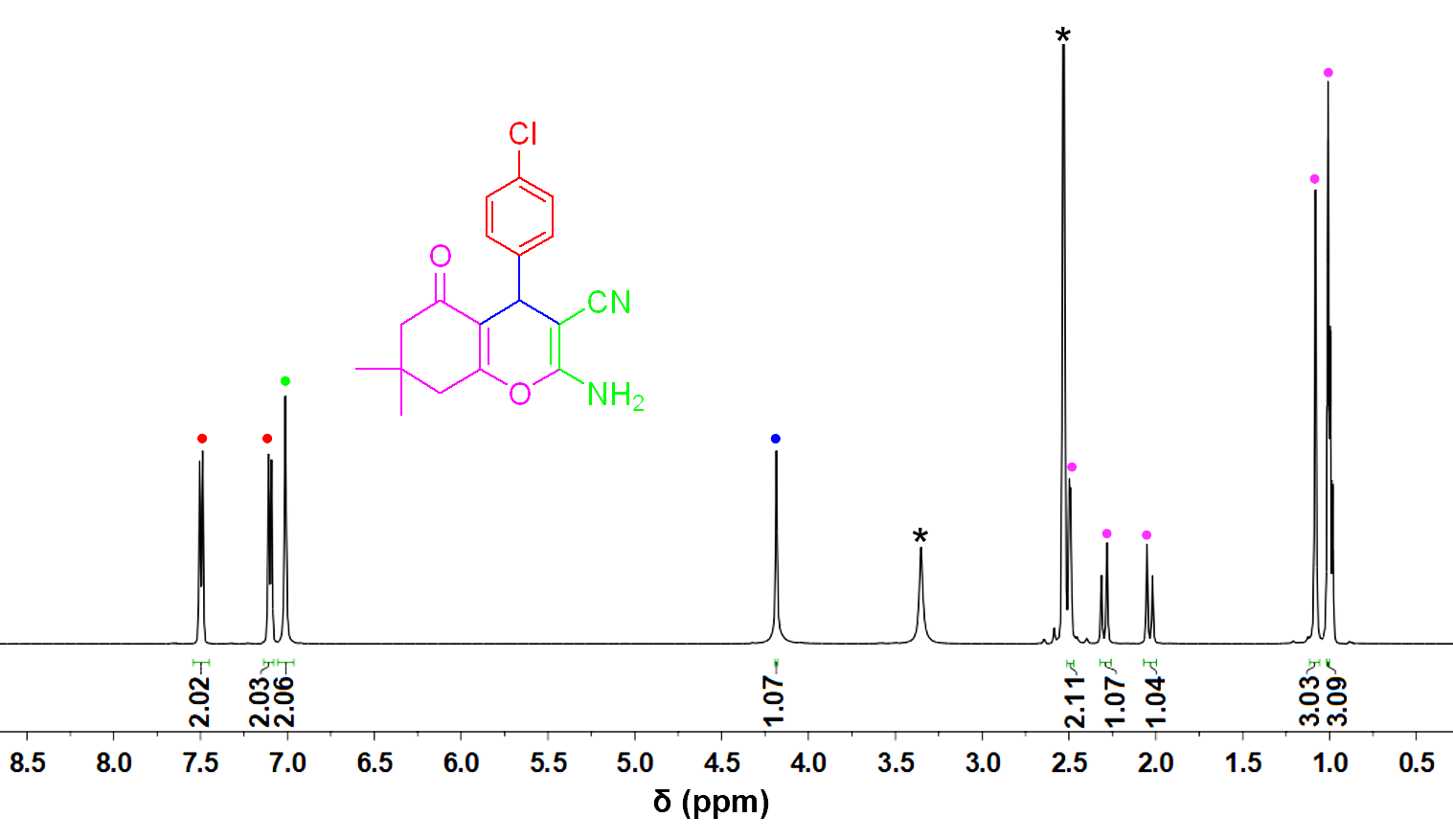


**Figure S4.** ^1^H NMR spectrum of compound **3** recorded in DMSO-d_6_.


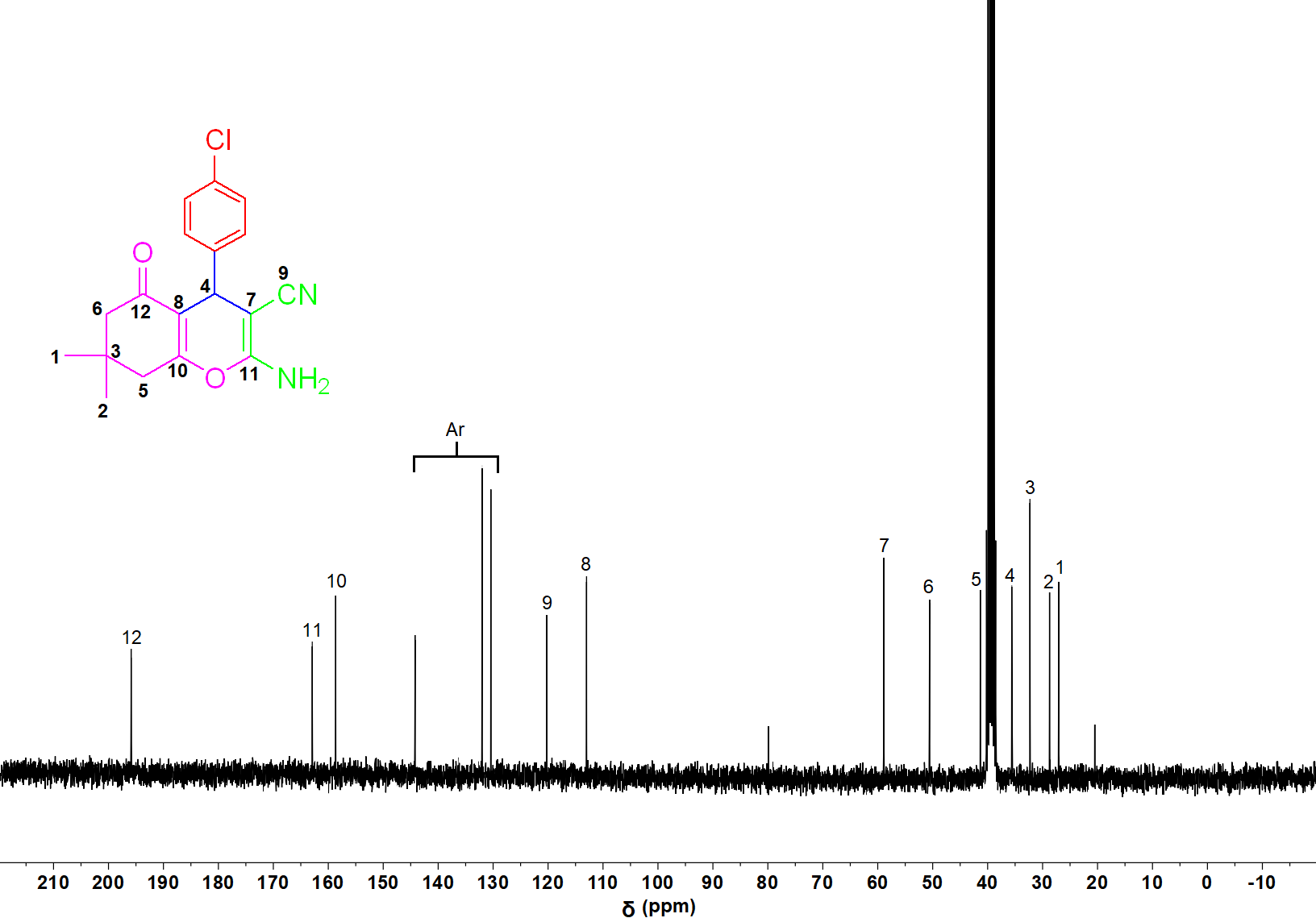


**Figure S5.** ^13^C NMR spectrum of compound **3** recorded in DMSO-d_6_.


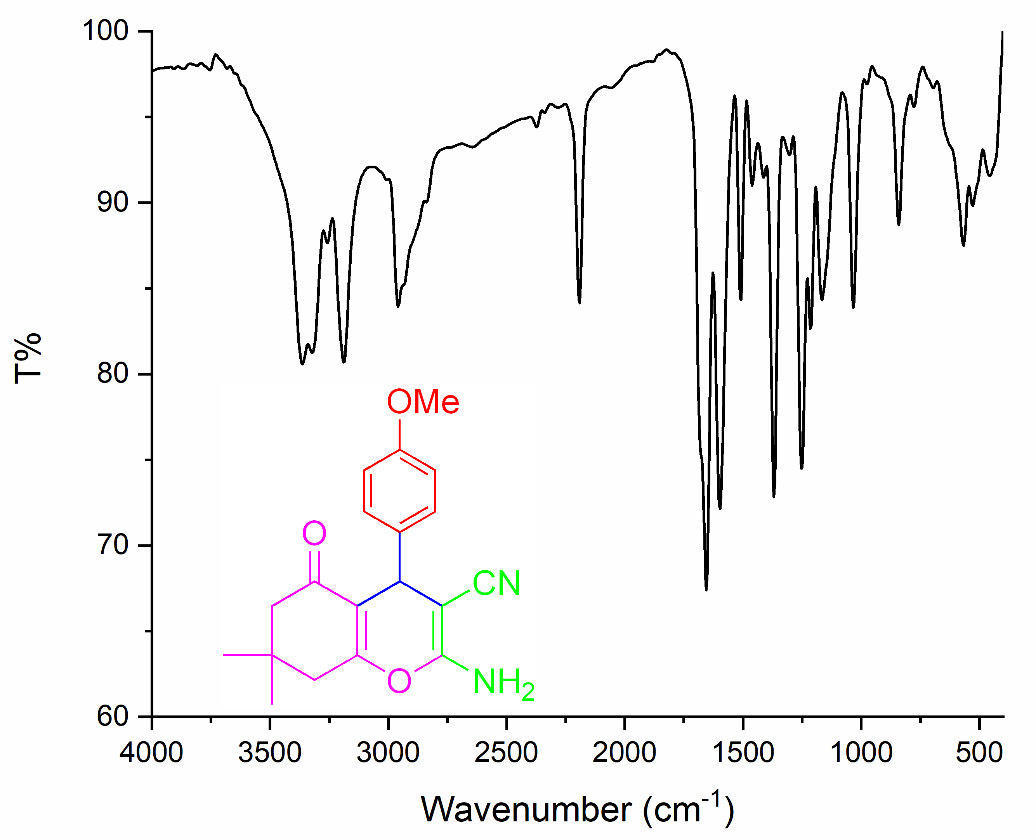


**Figure S6.** FT-IR spectra of compound **4**.


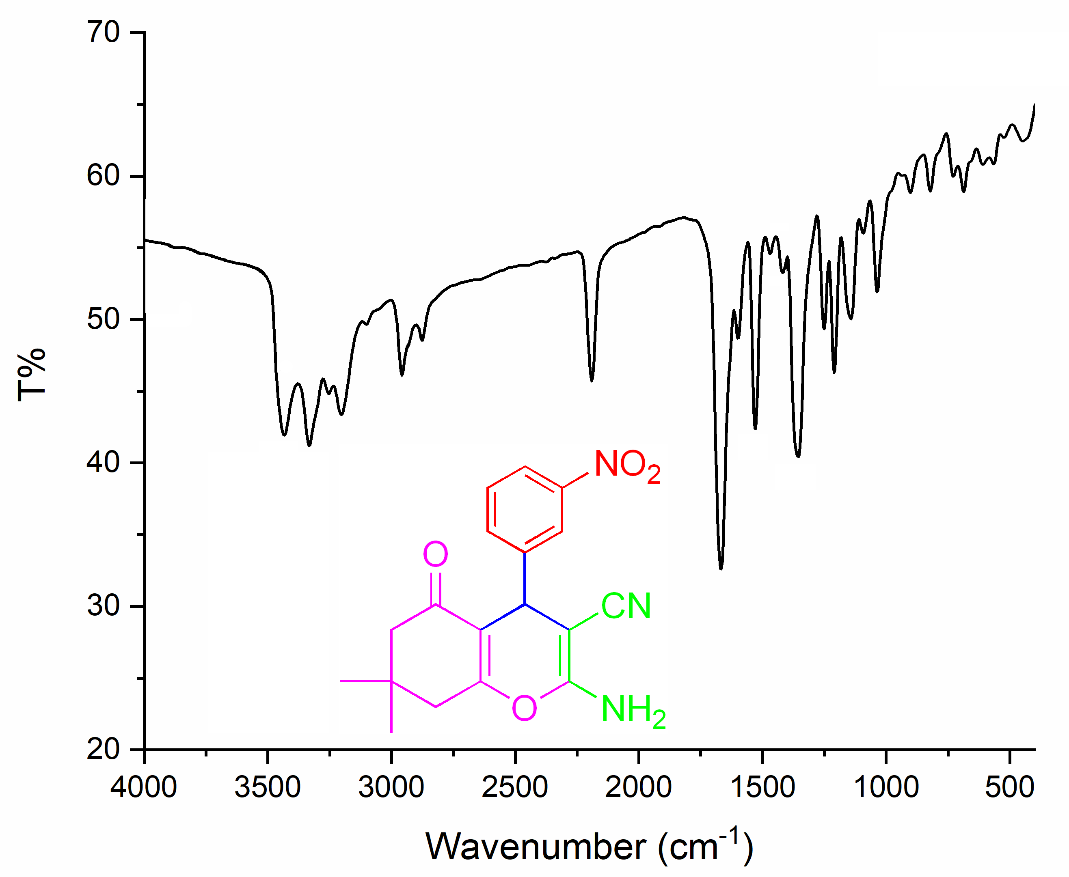


**Figure S7.** FT-IR spectra of compound **5**.


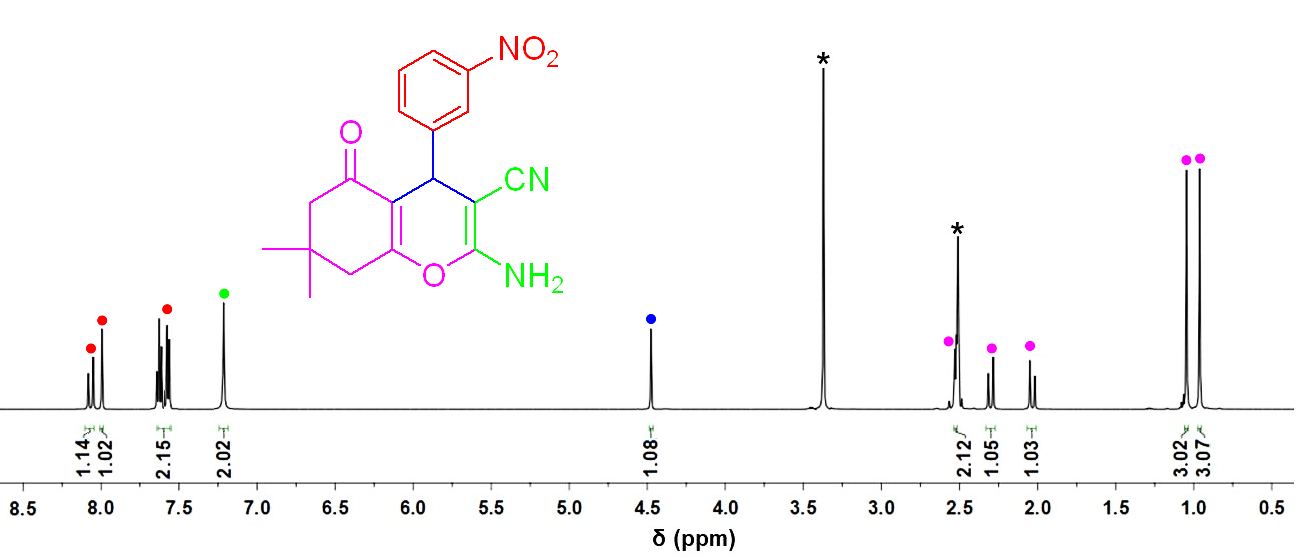


**Figure S8.** ^1^H NMR spectrum of compound **5** recorded in DMSO-d_6_.


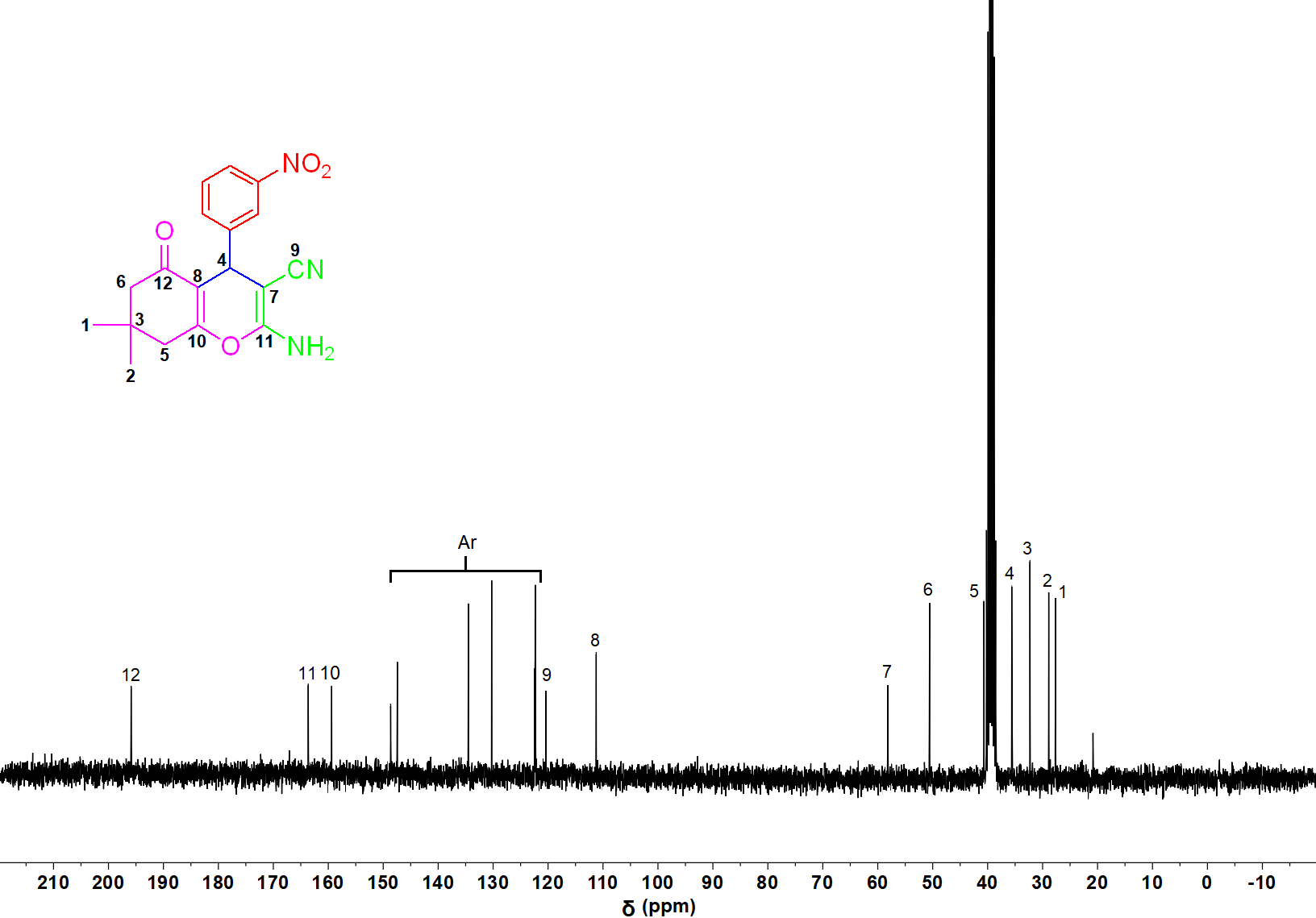


**Figure S9.** ^13^C NMR spectrum of compound **5** recorded in DMSO-d_6_.


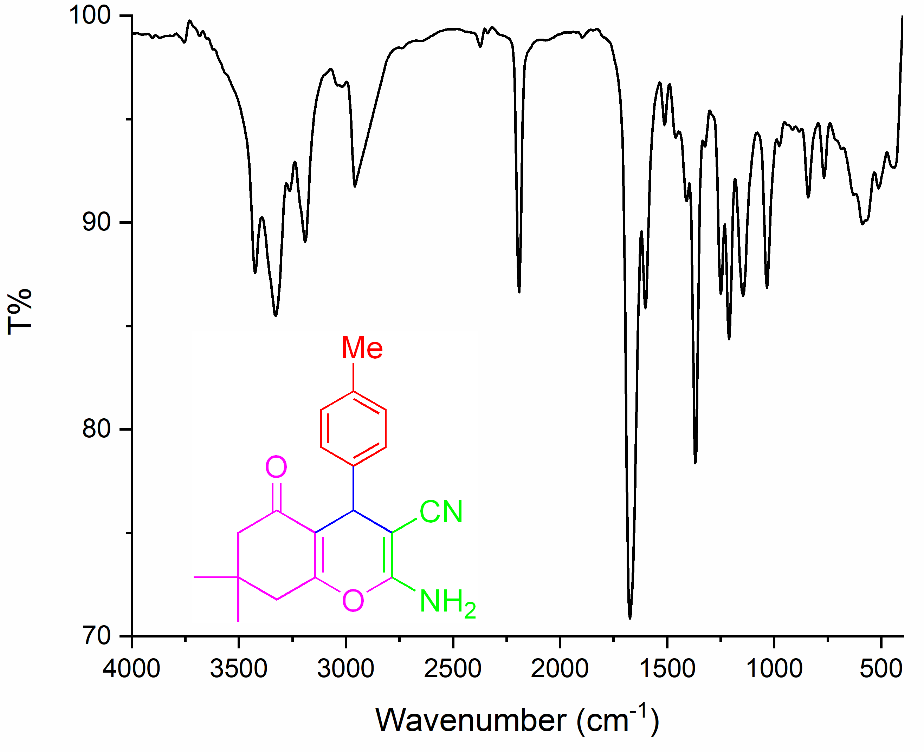


**Figure S10.** FT-IR spectra of compound **6**.


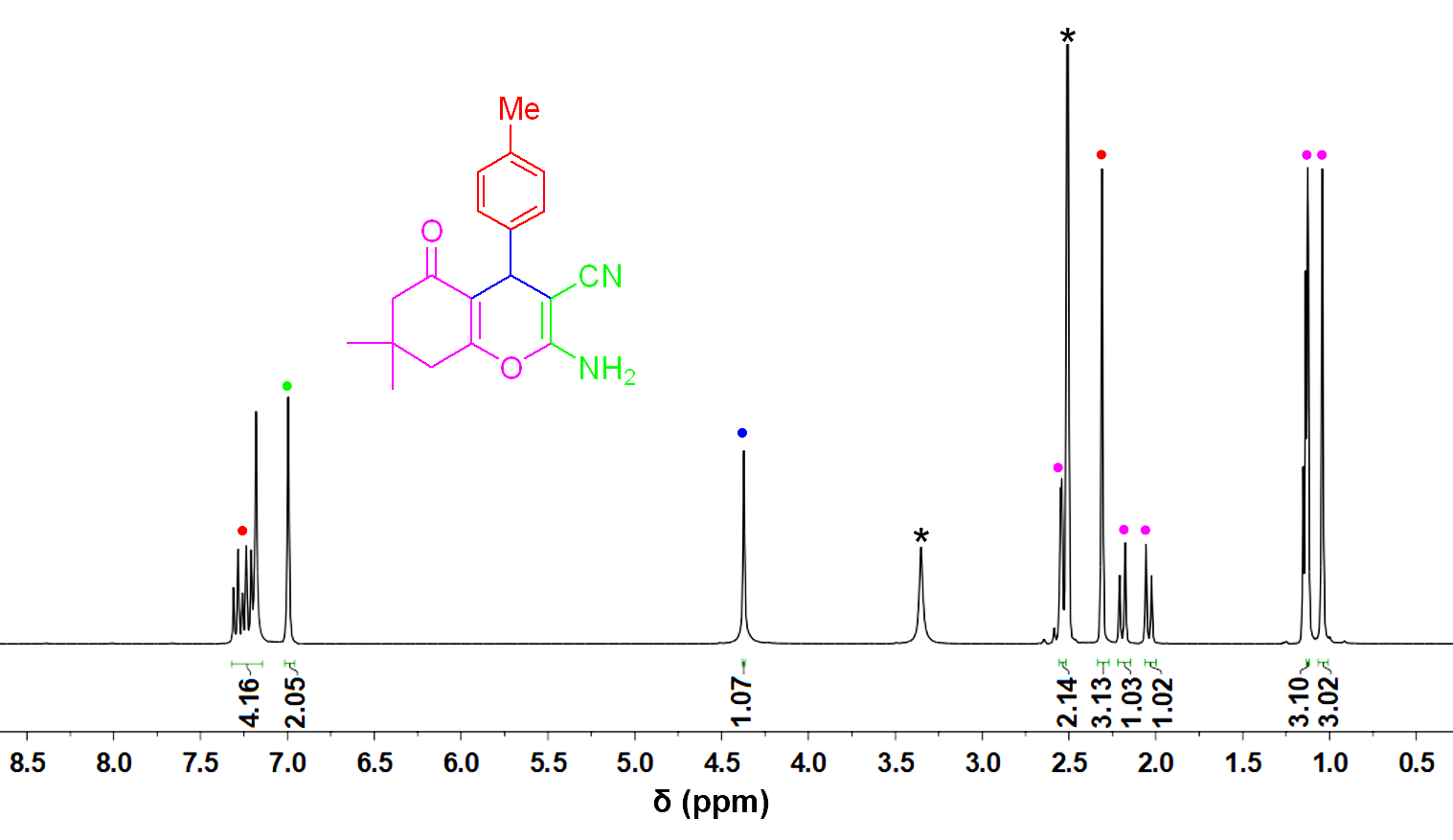


**Figure S11.** ^1^H NMR spectrum of compound **6** recorded in DMSO-d_6_.


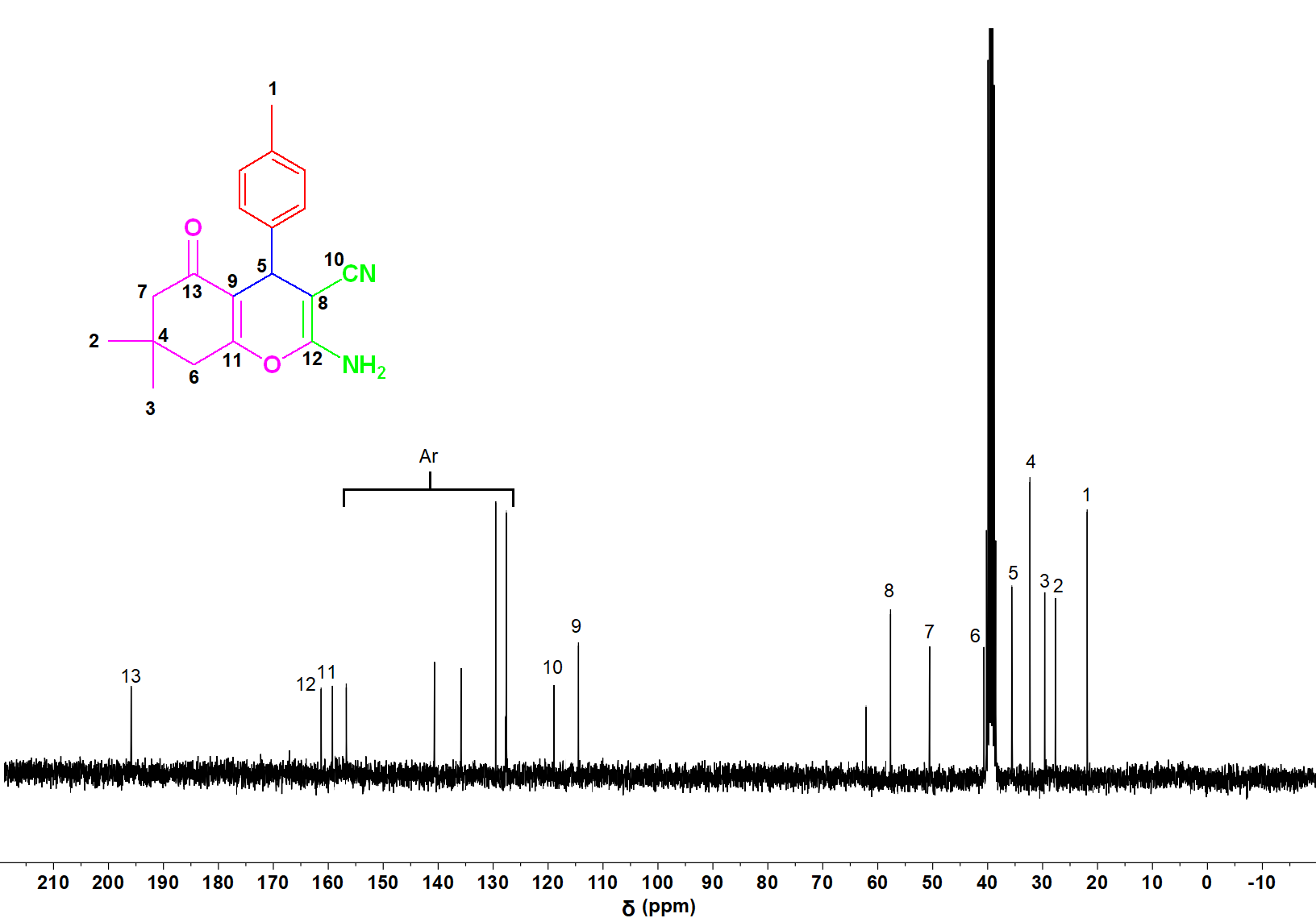


**Figure S12.** ^13^C NMR spectrum of compound **6** recorded in DMSO-d_6_.


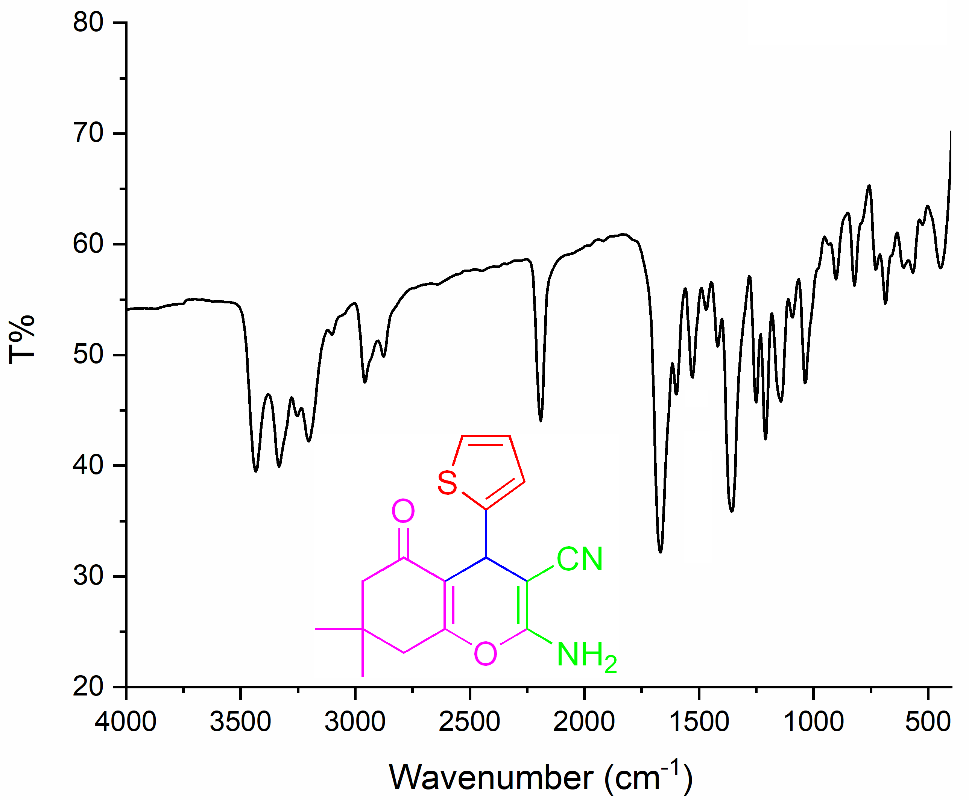


**Figure S13.** FT-IR spectra of compound **7**.


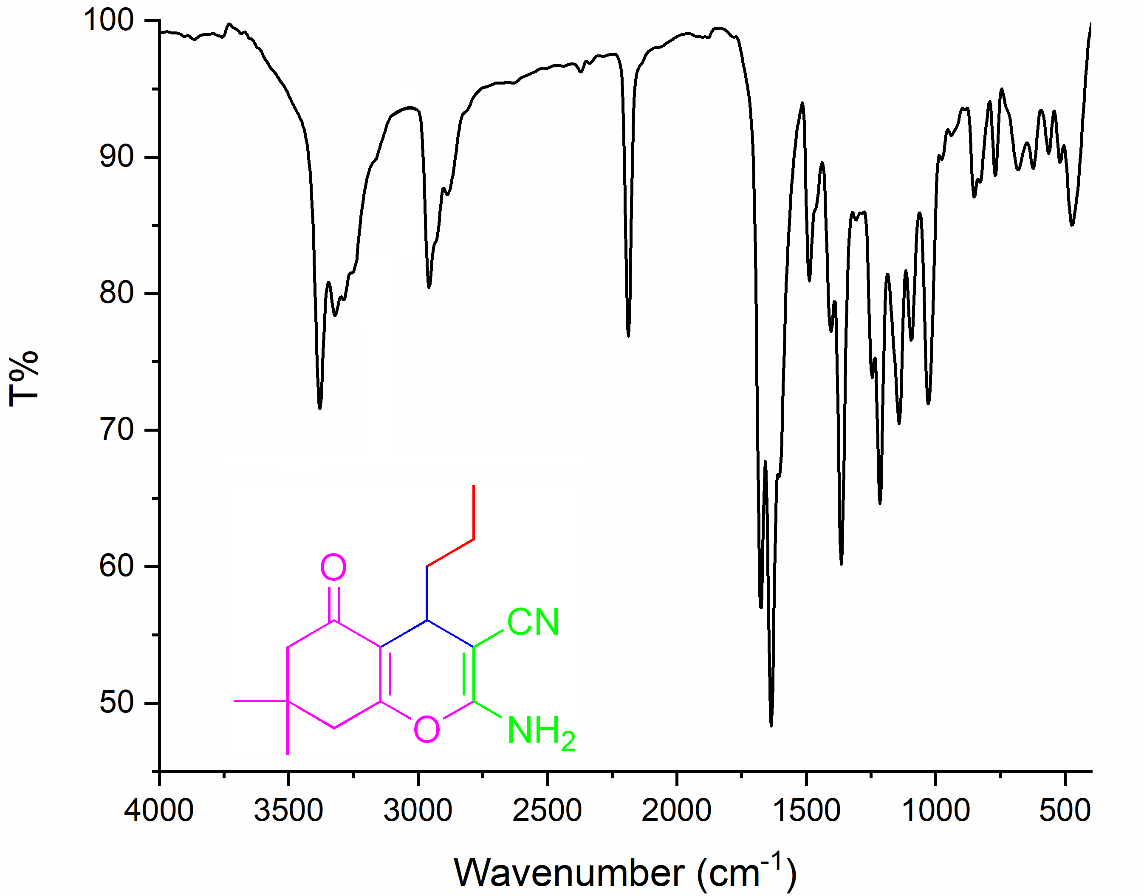


**Figure S14.** FT-IR spectra of compound **8**.
